# Developing an eVLP mRNA vaccine for respiratory syncytial virus with enhanced pre-fusion targeting humoral responses

**DOI:** 10.1128/jvi.01209-25

**Published:** 2025-09-30

**Authors:** Lei Sun, Mengting Huang, Simin Feng, Wei Zhang, Yun Quan, Ruyi Chen, Yupeng Yang, Haidong Xu, Wansheng Li, Qianyu Pan, Xinwen Chen, Danyang Zhang, Bin Yuan, Jincun Zhao, Zhongfang Wang, Jinzhong Lin, Wei Peng, Martin Ludlow, Qiong Zhang

**Affiliations:** 1 State Key Laboratory of Respiratory Disease, The First Affiliated Hospital of Guangzhou Medical University, Guangzhou Medical University 26468, Guangzhou, Guangdong, China; 2 Guangzhou National Laboratory, Guangzhou International Bio Island, Guangzhou, Guangdong, China; 3 Key Laboratory of Virology and Biosafety, Wuhan Institute of Virology, Chinese Academy of Sciences 74614 , Wuhan, Hubei, China; 4 State Key Laboratory of Genetic Engineering, School of Life Sciences, Zhongshan Hospital, Fudan University, Shanghai, China; 5 Shanghai Institute of Infectious Disease and Biosecurity, Fudan University, Shanghai, China; 6 Zhangjiang mRNA Innovation and Translation Center, Fudan University, Shanghai, China; 7 Research Center for Emerging Infections and Zoonoses (RIZ), University of Veterinary Medicine Hannover, Hannover, Germany; University of North Carolina at Chapel Hill, Chapel Hill, North Carolina, USA

**Keywords:** mRNA vaccine, respiratory syncytial virus, EABR, eVLPs, immune responses

## Abstract

Respiratory syncytial virus (RSV) is a leading cause of lower respiratory tract disease (LRTD) in infants, the elderly, and immunocompromised individuals. In this study, we leveraged the innovative EABR (ESCRT- and ALIX-binding region) vaccine technology to develop a pre-fusion (Pre-F)-based RSV mRNA vaccine (Pre-F-EABR) that encodes self-assembling enveloped virus-like particles (eVLPs). *In vitro* expression of Pre-F-EABR mRNA manifested pronounced Pre-F protein expression, particularly on cell membranes and in cell culture supernatant, compared to classical Pre-F and Pre-F-Ferritin nanoparticle (Pre-F-Fe) mRNAs, suggesting efficient eVLP self-assembly. Immunization of mice with lipid nanoparticle-encapsulated, nucleoside-modified mRNAs (mRNA-LNPs) demonstrated that the Pre-F-EABR vaccine elicited enhanced neutralizing activities and more robust cellular immunity when compared to Pre-F or Pre-F-Fe mRNA vaccines. Notably, the Pre-F-EABR mRNA vaccine induced a significantly higher level of protective pre-fusion-specific antibodies in contrast to post-fusion-targeting antibodies, dramatically reducing pulmonary viral load following RSV challenge in vaccinated mice. Furthermore, the Pre-F-EABR mRNA vaccine demonstrated significantly improved efficacy against the contemporary clinical ON1 and BA9 isolates. Overall, these findings demonstrate that the Pre-F-EABR mRNA vaccine induces more robust immune responses, highlighting its potential in preventing RSV infection in vulnerable populations.

**IMPORTANCE:**

Respiratory syncytial virus (RSV) is a major cause of lower respiratory tract disease in infants, the elderly, and immunocompromised individuals. It is well-established that enhancing the neutralizing antibody levels and Th1-biased cellular immune responses can potentially improve the efficacy and safety of RSV vaccines. In this study, we developed an RSV pre-fusion protein-based mRNA vaccine that encodes self-assembling enveloped virus-like particles. Mice immunized with this vaccine showed significantly enhanced pre-fusion protein-targeted humoral responses and improved protection against RSV infection compared to the conventional RSV mRNA vaccine. Additionally, this vaccine demonstrated a considerably stronger neutralizing ability against contemporary clinical RSV isolates and induced more robust Th1-biased cellular immune responses, suggesting its potential as a promising RSV vaccine candidate.

## INTRODUCTION

Respiratory syncytial virus (RSV), a single-stranded negative-sense RNA virus, is a worldwide leading cause of severe lower respiratory tract disease (LRTD) among infants, the elderly, and immunocompromised individuals. As one of the primary contributors to childhood (≤5 years of age) morbidity and mortality (1–5), RSV infects nearly every child by the age of two, with recurrent infections occurring throughout life (1–5). Current global estimates indicate approximately 33 million annual cases of RSV-associated LRTD in young children, including 3 million hospitalizations (1, 3, 4).

While vaccination remains the most effective strategy for preventing RSV infection and severe LRTD (6), vaccine development has long been hampered by concerns over potential vaccine-associated enhanced respiratory disease (VAERD) first observed in RSV-naive infants vaccinated with a formalin-inactivated RSV (FI-RSV) in the 1960s (7). Although the precise mechanisms underlying VAERD remain incompletely characterized, current evidence implicates that a T helper (Th) 2-biased CD4+ T-cell response, poor induction of neutralizing antibodies, a lack of CD8+ cytotoxic T-cell responses, and pulmonary eosinophilia following RSV challenge were the main attributes (8–10). Consequently, optimal RSV vaccine candidates should promote the induction of Th1-polarized cellular immunity coupled with superior neutralizing activities to ensure comprehensive protection (6, 11).

The RSV fusion glycoprotein (F) serves as the primary target of neutralizing antibodies in human sera (12, 13) and represents the most promising antigen for RSV vaccine development. This metastable protein mediates the fusion of viral particles to the host cell membranes through structural rearrangement from its prefusion (Pre-F) conformation to the more stable postfusion (Post-F) state (14–16). Although clinical trials of RSV Post-F-based vaccines demonstrated only modest protective efficacy (17), Pre-F antigen formulations have consistently shown enhanced immunogenicity and superior protection in clinical evaluations (15, 18–20), primarily due to their capacity to elicit highly potent neutralizing antibodies and memory B cell responses targeting Pre-F-specific antigenic sites (Ø and V) (6, 12, 15, 21). The clinical translation of Pre-F vaccines has been hindered by the protein’s conformational instability (6). Structure-based stabilization strategies, particularly through rational design of disulfide bonds and cavity-filling mutations (14, 15, 22), have overcome this limitation and revolutionized RSV vaccine development (6, 19, 20, 23). DS-Cav1, the most prominent stabilized Pre-F prototype, elicits high-affinity antibodies with strong neutralizing ability in mice, non-human primates, and seropositive adults (14, 15, 21, 24, 25). These advances have paved the way for three approved RSV vaccines, including GSK’s *Arexvy*, Moderna’s *mResvia*, and Pfizer’s *Abrysvo* (18, 19, 26), as well as other vaccine candidates currently under preclinical and clinical development (23, 27, 28).

The widespread deployment of COVID-19 mRNA vaccines developed by Moderna and Pfizer/BioNTech has demonstrated the safety, efficacy, and reliability of lipid nanoparticle (LNP)-encapsulated, nucleoside-modified mRNA vaccines (29–31). Unlike conventional vaccines, mRNA-based vaccines leverage host cellular machinery to produce antigens, thereby activating specific and efficient humoral immune responses and T-cell immunity (32). This attribute is particularly advantageous for RSV vaccine development, as mRNA platforms preferentially induce robust humoral responses and Th1-skewed cellular immunity (20, 33). Accordingly, a single dose of the mRNA-based RSV vaccine *mResvia* conferred an 83.7% efficacy against RSV-associated LRTD in adults aged 60 and older over 3.7 months (20). However, the efficacy of the vaccine against LRTD declined to ~50% at 18 months, underscoring the need to reassess the current RSV mRNA vaccine designs for durable protection in vulnerable populations.

A recent study introduced an innovative mRNA vaccine strategy that integrates the mRNA vaccine platform with nanoparticle technology by incorporating an ESCRT (endosomal sorting complex required for transport)- and ALIX (ALG-2-interacting protein X)-binding region (EABR) into the cytoplasmic tail of the severe acute respiratory syndrome coronavirus 2 spike protein (34). This Spike-EABR mRNA vaccine leverages host cell ESCRT machinery to assemble enveloped virus-like particles (eVLPs) that display densely arrayed spike proteins on their surface, mimicking the natural budding process of enveloped viruses. Compared to conventional COVID-19 mRNA vaccines, the Spike-EABR construct induced enhanced neutralizing activities and more potent T-cell immune responses in preclinical models. However, the broader applicability of this platform—particularly for other high-priority pathogens like RSV—remains unexplored.

Herein, we employed the eVLP vaccine technology to develop a novel RSV Pre-F-EABR mRNA vaccine. Comparative immunization studies in mice demonstrated that lipid nanoparticle-encapsulated, nucleoside-modified Pre-F-EABR mRNA elicited enhanced neutralizing activities and more robust T-cell responses when compared to both the mRNA vaccine encoding DS-Cav1 (Pre-F) and the Pre-F-Ferritin nanoparticle mRNA vaccine. Notably, the Pre-F-EABR mRNA vaccine elicited significantly higher titers of protective Pre-F-specific antibodies in contrast to Post-F-directed antibodies, resulting in a dramatic reduction in pulmonary viral loads following RSV challenge in vaccinated mice. Moreover, the Pre-F-EABR vaccine exhibited significantly improved breadth of protection against contemporary clinical RSV-A (ON1 genotype) and RSV-B (BA9 genotype) strains. Overall, the Pre-F-EABR mRNA vaccine induces robust immune responses and holds great potential in preventing RSV infection.

## RESULTS

### mRNA vaccine design and the *in vitro* expression characterization

**Fig 1:**
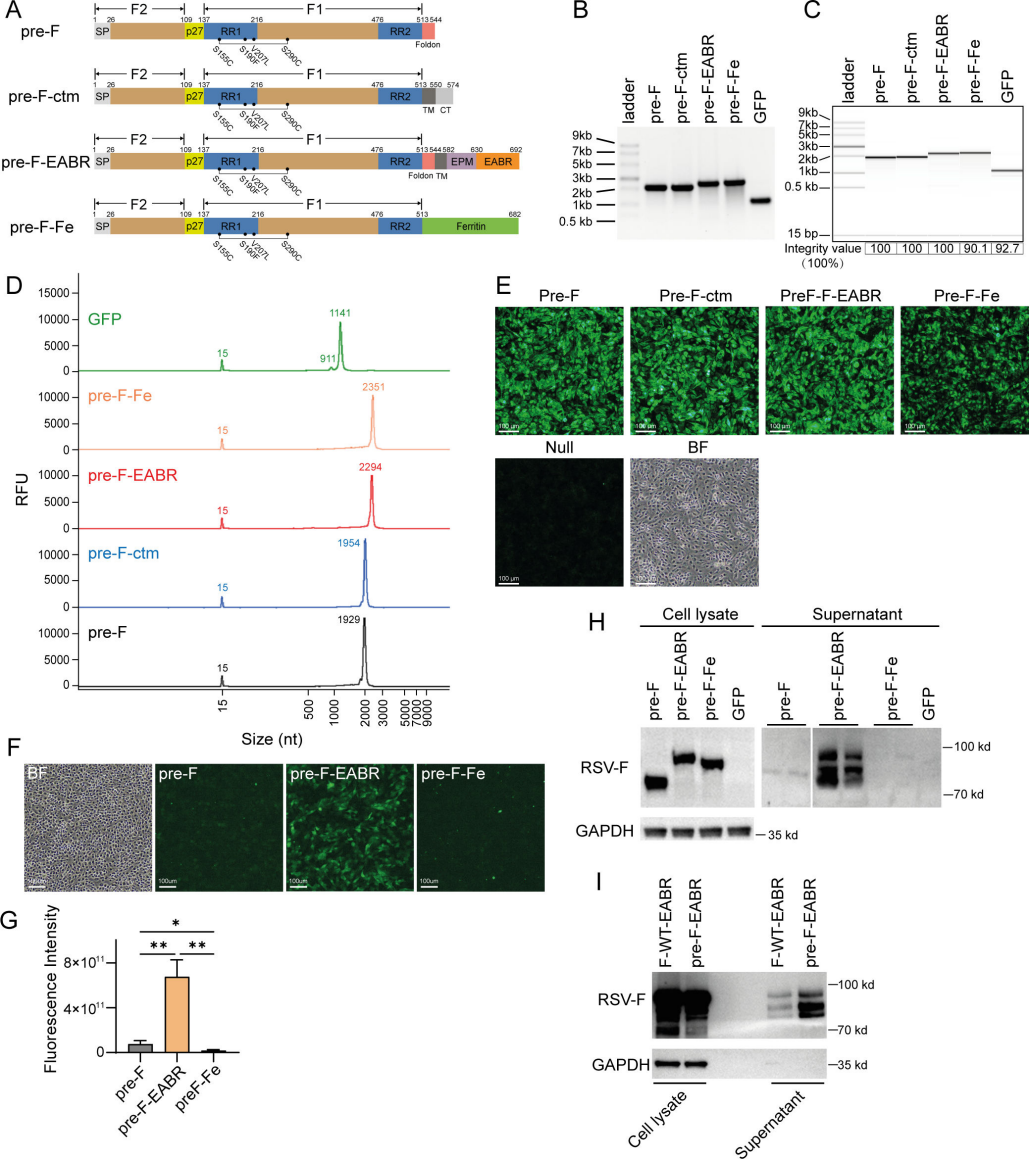
The design and *in vitro* characterization of mRNA vaccines. (**A**) Schematic representation of mRNA constructs expressing RSV prefusion F protein. The prefusion-stabilized F protein was designed based on the DS-Cav1 structure, using RSV F from strain A2. Key domains are labeled: signal peptide (SP), p27 peptide, fusion peptide (FP), refolding regions 1 and 2 (RR1, RR2), transmembrane domain (TM), and cytoplasmic tail (CT). Prefusion-stabilizing mutations are indicated by solid circles (•), and the disulfide bond is indicated with a bracket. The T4-fibritin foldon domain mediates trimerization. Other labeled segments include the ferritin domain from *H. pylori*, an EPM, and an ESCRT- and ALIX-binding region (EABR) (34). (**B**) The designed constructs were *in vitro* transcribed and verified via agarose gel electrophoresis. (**C**) The integrity of mRNAs was analyzed by capillary gel electrophoresis (integrity values [%] labeled below). (**D**) Capillary gel electropherograms are presented, with the size (nt) of fragments corresponding to major peaks annotated numerically. (**E**) Immunofluorescence detection of Pre-F protein expression in BHK-21 cells 24 h post-transfection of mRNAs with RSV Pre-F specific monoclonal antibody D25. (**F**) Immunofluorescence detection of membrane-localized Pre-F protein in BHK-21 cells 24 h post-transfection of mRNAs, with (**G**) quantitative analysis by high-content fluorescence scanning. (**H**) eVLPs in HEK293T cell supernatants were detected by western blot 72 h following mRNA transfection. (**I**) mRNAs encoding the pre-F-EABR were transfected into HEK293F cells, and eVLPs in cell culture supernatants were concentrated and detected by western blot analysis. For western blot analyses (panels H and I), RSV F protein was detected using motavizumab (anti-RSV F monoclonal antibody), with anti-GAPDH as a loading control. Images shown in panel H are representative of results from at least three independent experiments. The data in (**G**) represent mean ± standard deviation (SD) (*n* = 3) and statistical comparisons were estimated by two-tailed unpaired *t*-test (*P ≤ 0.05; **P ≤ 0.01).

A recent study has demonstrated the ability of the “EABR technology” to elicit potent humoral and T-cell responses (34), prompting speculation about its applicability to vaccines against other life-threatening pathogens, such as RSV, via the production of self-assembled eVLPs. Therefore, we designed a Pre-F-EABR mRNA vaccine using a strategy similar to the Spike-EABR vaccine: the EABR segment, derived from the human CEP55 protein, was fused to the C terminus of the prefusion stabilized DS-Cav1 construct (Fig. 1A). To prevent coated pit localization and endocytosis, an endocytosis prevention motif (EPM) derived from the murine Fc gamma receptor FcgRII-B1 cytoplasmic tail was inserted upstream of the EABR sequence (34). Vaccinations with DS-Cav1 or its corresponding mRNA have consistently elicited robust neutralizing antibody and T-cell responses in both animal models and human clinical trials (15, 21, 24, 28). For comparison, we also included the pre-F-ctm vaccine design (a full-length RSV F protein containing the four point mutations present in DS-Cav1) (33). Additionally, an mRNA construct encoding a DS-Cav1 displaying nanoparticle was formulated for comparative analysis by introducing the ferritin sequence from *Helicobacter pylori* at the C terminus of DS-Cav1 (Pre-F-Fe) (Fig. 1A). Swanson et al. previously validated the self-assembly of the ferritin nanoparticle by *in vitro* overexpression of the Pre-F-Fe construct (35).

Following synthesis, mRNA integrity was evaluated by TBE RNA gel electrophoresis (Fig. 1B) and capillary gel electrophoresis (CGE) (Fig. 1C and D). CGE analysis revealed single, homogeneous peaks corresponding to the expected sizes for all RSV mRNA constructs, with measured integrity values >90% for all constructs (Fig. 1C). The translational efficiency of the synthesized mRNAs was assessed by transfection of mRNAs into BHK-21 cells. Intracellular antigen expression was analyzed at 24 h post-transfection via indirect immunofluorescence staining using the Pre-F-specific antibody D25, followed by a fluorophore-conjugated secondary antibody (Fig. 1E). Robust and comparable protein expression was demonstrated across all constructs. Beyond intracellular expression, cell surface antigen presentation was evaluated by staining with the same antibody (D25). Unlike the intracellular expression pattern, Pre-F-EABR mRNA demonstrated significantly enhanced membrane localization (>8-fold enrichment) compared with other constructs (Fig. 1F and G). Notably, Pre-F-Fe was completely absent from the cell surface. The significant enrichment of Pre-F-EABR on the cell membrane aligns with our vaccine design strategy and enables the assembly and budding of eVLPs that can activate immune cells. The release of eVLPs into culture supernatants of HEK293T and HEK293F cells was evaluated by western blot analysis following the ultracentrifugation of transfected cell supernatants on a 20% sucrose cushion to concentrate eVLPs (34). In accordance with the cell surface expression results, Pre-F and Pre-F-Fe transfections did not generate detectable eVLPs in supernatants, whereas eVLPs were readily detected in supernatants from Pre-F-EABR mRNA-transfected cells (Fig. 1H and I). Collectively, the mRNA expression results demonstrate that the mRNA-encoded Pre-F-EABR construct enables the pronounced expression of Pre-F protein, the enrichment of antigens on cell surfaces, and the release of eVLPs.

**Fig 2:**
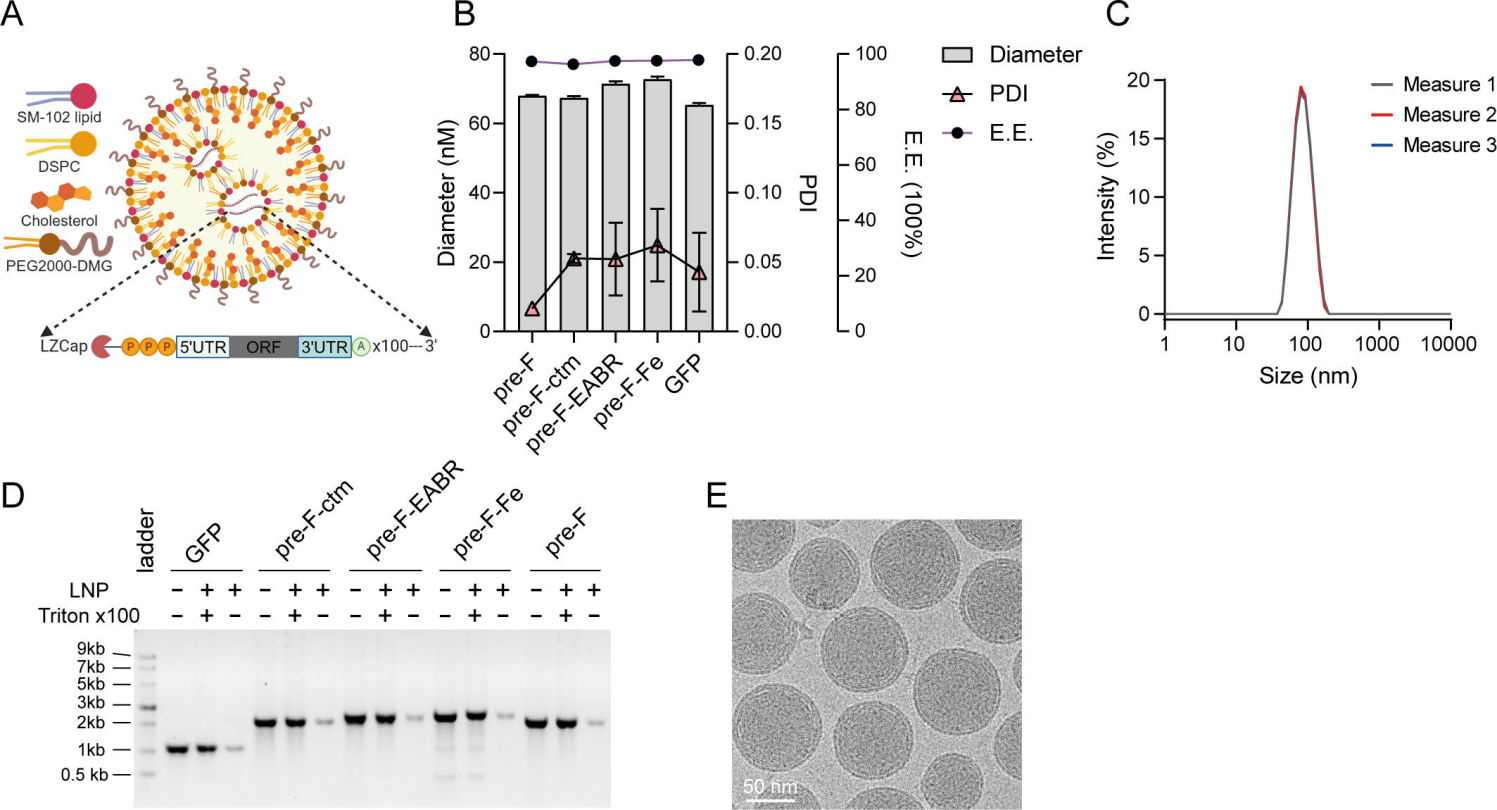
Characterization of prepared mRNA-LNPs. (**A**) Schematic illustration of the formulated mRNA-LNP, as described in Materials and Methods. (**B**) Physicochemical properties of mRNA-LNPs, including Z-average size, PDI measured by dynamic light scattering (DLS), and encapsulation efficiency (E.E.) determined by RiboGreen RNA assay. (**C**) Particle size distribution profile of the formulated LNPs obtained by DLS. (**D**) Assessment of mRNA encapsulation efficiency via agarose gel electrophoresis following treatment with Triton X-100. (**E**) cryo-EM image showing the morphology of the formulated LNPs. Data in (**B**) represent mean ± SD (*n* = 3).

Prior to evaluating the vaccine efficacy in murine models, we formulated Pre-F, Pre-F-ctm, Pre-F-EABR, and Pre-F-Fe mRNAs using the classical SM102-based four-component LNPs (Fig. 2A). The formulated mRNA-LNPs exhibited uniform hydrodynamic diameters ranging from 60 to 70 nm with a polydispersity index (PDI) ≤0.07 (Fig. 2B and C). Cryo-electron microscopy (Cryo-EM) analysis of the mRNA-LNP confirmed the formation of uniformly arrayed spherical particles with regular morphology (Fig. 2E). Additionally, all mRNA-LNPs manifested >90% encapsulation efficiency as quantified by RiboGreen assay and verified through agarose gel electrophoresis of Triton X-100-treated versus untreated mRNA-LNPs (Fig. 2B and D). Overall, these comprehensive characterizations indicate proper preparation of mRNA-LNPs with optimal physicochemical properties and biological activity.

### Pre-F-EABR mRNA vaccine induced enhanced Pre-F-specific IgG antibody responses in murine models

**Fig 3:**
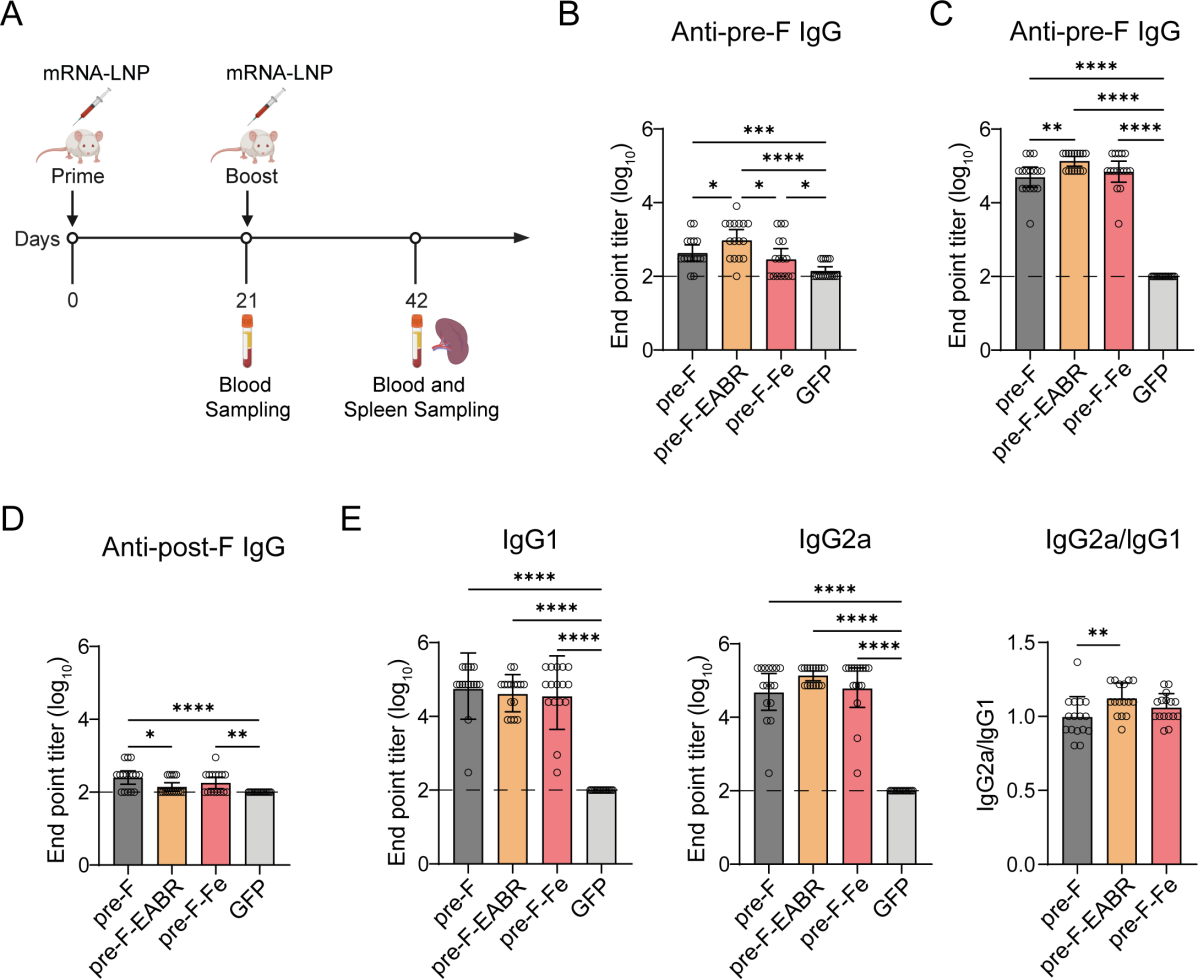
Characterization of humoral immune responses elicited by mRNA-LNPs in mice. (**A**) Immunization scheme: female BALB/c (*n* = 16 per group) mice were immunized I.M. with 5 µg mRNA-LNP vaccines in a prime-boost regimen at day 0 and day 21. Serum was collected on days 21 (post-prime) and 42 (post-boost). RSV pre-F-specific binding antibody titers post-prime (day 21) (**B**) and post-boost (day 42) (**C**), measured by ELISA. (**D**) RSV post-F binding antibody titers post-boost were assayed as well. (**E**) IgG1 and IgG2a subclass antibody titers against RSV pre-F were determined, and the ratio of IgG2a/IgG1 was calculated to signify a Th2 or Th1 type response. The ELISA data represent the geometric mean with a 95% confidence interval for each group. The limit of detection is shown as a dotted line. Statistical significance was calculated by a two-tailed unpaired *t*-test. *P < 0.05; **P < 0.01; ***P < 0.001; ****P < 0.0001. Graphs are representative of three independent experiments.

To characterize the dose-response relationship of the mRNA vaccine, female BALB/c mice were intramuscularly (I.M.) immunized with escalating doses (0.1, 1, and 5 µg) of the pre-F-EABR mRNA vaccine in a prime-boost regimen with a 3-week interval (Fig. 3A). Serum samples collected 3 weeks post-immunization were analyzed for Pre-F protein-specific IgG titers by enzyme-linked immunosorbent assay (ELISA) (Fig. S1A and C). Both prime and boost vaccinations induced dose-dependent increases in Pre-F-specific antibody titers. Neutralization assays against multiple RSV subtypes (A2, ON1, and BA9) revealed similarly dose-dependent enhancement of neutralizing antibody activity in both prime and boost sera (Fig. S1B and D). Notably, the 5 µg dose consistently generated the highest Pre-F-specific IgG titers and most potent neutralizing responses across all tested subtypes, supporting its selection for subsequent immunization studies.

To evaluate mRNA vaccine-elicited humoral responses, serum samples collected post-prime and post-boost immunization (5 µg dose) were analyzed by ELISA for Pre-F- and Post-F-specific antibody responses (Fig. 3B through E; Fig. S2A and B). Following the prime immunization, all mRNA-LNP vaccines elicited moderate levels of Pre-F-specific binding antibodies; however, the titers induced by the Pre-F-EABR were significantly higher (2.2- to 5.7-fold increase) than those produced by other formulations (Fig. 3B; Fig. S2A). Following the boost immunization, all mRNA-LNP vaccines elicited robust Pre-F-specific binding antibody responses but low Post-F-specific binding antibody titers, confirming that *in vivo*-expressed antigens predominantly maintained the prefusion conformation (Fig. 3C and D; Fig. S2B). Remarkably, the Pre-F-EABR construct consistently elicited more favorable antibody responses, achieving 2.0- to 6.1-fold higher Pre-F-specific binding antibody titers than other mRNA-LNPs (Fig. 3C; Fig. S2B) while maintaining the lowest Post-F-binding antibody titers (1.9-fold lower than Pre-F and 1.3-fold lower than Pre-F-Fe mRNA-LNPs; Fig. 3D). This superiority extended to the 1 µg dose, where Pre-F-EABR vaccination induced 3.5- to 4.9-fold higher Pre-F-specific antibody titers after prime immunization and 8.7- to 17.8-fold higher titers after boost immunization compared to other constructs (Fig. S2C and D). Additionally, Pre-F-EABR at 1 µg induced 4.8- and 5.8-fold higher Pre-F-specific antibody titers than Pfizer’s subunit vaccine (18, 36, 37) after prime and boost immunization, respectively. These results suggest that the EABR segment may enhance the stability of the Pre-F conformation, thereby enhancing the antibody responses.

Furthermore, we assessed Th1/Th2 immune polarization by determining Pre-F-specific IgG1 and IgG2a subclasses in post-boost sera (Fig. 3E). In mice, type-1 cytokine interferon γ (IFN-γ) promotes IgG2a secretion while inhibiting IgG1, whereas type-2 cytokine interleukin-4 (IL-4) promotes IgG1 secretion while inhibiting IgG2a (38). As depicted, all three mRNAs elicited high titers of both IgG2a and IgG1 Pre-F-specific binding antibodies compared to the control, resulting in IgG2a/IgG1 ratios approaching 1.0, indicative of a balanced Th1/Th2 response. Nevertheless, the Pre-F-EABR group displayed a modest but statistically significant increase in IgG2a/IgG1 ratio (*P* < 0.01 by two-tailed unpaired *t*-test) compared to the Pre-F mRNA-LNP, suggesting that Pre-F-EABR may have greater potential in stimulating Th1-skewed immune response.

### Pre-F-EABR mRNA vaccine induced elevated cross-neutralizing antibody responses against diverse viral strains

**Fig 4:**
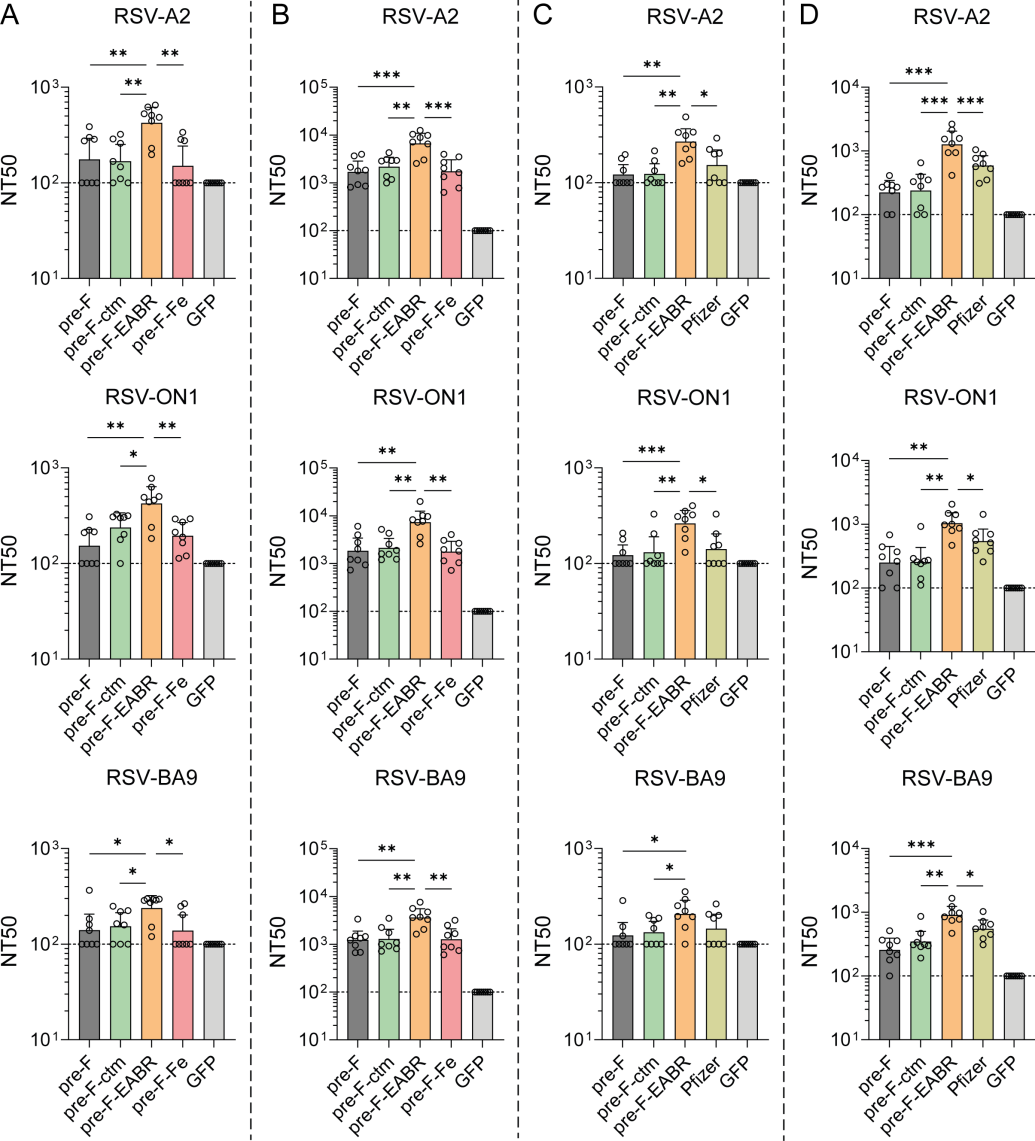
Neutralizing antibody responses induced by 1 and 5 µg mRNA-LNPs in BALB/c mice. Female BALB/c mice (*n* = 8/group) were immunized I.M. with 1 or 5 µg mRNA-LNPs in a prime-boost regimen (days 0 and 21, see Fig. 3A). (**A, B**) Neutralization activity of post-prime (day 21, **A**) and post-boost (day 42, **B**) sera from mice immunized with 5 µg mRNA-LNPs, tested against RSV-A2 (top panel), RSV-ON1-GFP (middle panel), and RSV-BA9-GFP (bottom panel) strains. (**C, D**) Neutralization activity of post-prime (day 21, **C**) and post-boost (day 42, **D**) sera from mice immunized with 1 µg mRNA-LNPs, tested against the same RSV strains. NT50 values (reciprocal serum dilution achieving 50% neutralization) were determined by four-parameter sigmoidal curve fitting. Data represent geometric mean ± 95% CI (dotted line indicates limit of detection). Statistical significance was calculated by two-tailed unpaired *t*-test (*P < 0.05; **P < 0.01; ***P < 0.001; ****P < 0.0001).

Beyond F protein-targeting IgG responses, neutralization profiles against multiple RSV subtypes—including the prototype RSV A2 strain and two contemporary clinical isolates representing current circulating ON1 (RSV-A-0594 strain) and BA9 (RSV-B-9671 strain) genotypes were comprehensively evaluated in mice immunized with 1 and 5 µg doses of mRNA-LNPs (Fig. 4). Primary immunization with either dose elicited modest yet detectable neutralizing antibody titers against all tested RSV strains (Fig. 4A and C). Notably, the Pre-F-EABR vaccine consistently elicited stronger neutralizing activities, exhibiting statistically significant higher titers against all tested RSV strains—RSV A2 (2.2- to 2.6-fold), RSV ON1 (1.8- to 2.7-fold), and RSV BA9 (1.4- to 1.7-fold)—compared to other vaccine formulations at both dose levels. Booster immunization with 5 µg mRNA-LNPs significantly boosted the neutralizing antibody responses (Fig. 4B). In line with primary immunization results, the Pre-F-EABR mRNA-LNP consistently outperformed other vaccine candidates in stimulating neutralizing antibody responses across all tested strains. Against the RSV A2 strain, geometric mean neutralization titers were 3.8-, 3.0-, and 3.7-fold higher than those elicited by Pre-F, Pre-F-ctm, and Pre-F-Fe vaccines, respectively (Fig. 4B, top panel). This enhanced neutralization capacity extended to contemporary clinical isolates, with Pre-F-EABR showing 3.7-, 3.5-, and 4.2-fold increase against RSV subtype ON1 (39) and 2.9-, 2.7-, and 2.9-fold elevations against RSV subtype BA9 (39) compared to the control vaccines (Pre-F, Pre-F-ctm, and Pre-F-Fe constructs, respectively) (Fig. 4B, middle and bottom panels). While immunization with a 1 µg dose of mRNA vaccines generally induced substantially lower neutralization titers compared to the 5 µg dose, the Pre-F-EABR mRNA vaccine demonstrated more pronounced advantages when compared to Pre-F and Pre-F-ctm mRNA vaccines (Fig. 4D). At this reduced antigen dose, Pre-F-EABR demonstrated 5.8- and 4.9-fold higher neutralization titers against RSV A2 (top panel), 3.7- and 3.6-fold greater activity against RSV ON1 (middle panel), and 3.4- and 2.5-fold increased neutralization capacity against RSV BA9 subtypes (Fig. 4D, bottom panel) compared to the Pre-F and Pre-F-ctm vaccines, respectively. Notably, neutralization assays further demonstrated that the Pre-F-EABR mRNA vaccine elicited neutralization antibody responses that were 1.5- to 2.2-fold stronger than those induced by the Pfizer subunit vaccine across all tested RSV subtypes (Fig. 4C and D), consistent with the observed Pre-F-specific antibody titers (Fig. S2C and D). No significant differences in serological immune responses were observed among the Pre-F, Pre-F-ctm, and Pre-F-Fe constructs.

Collectively, these findings demonstrate that mRNA-LNP-generated Pre-F-EABR eVLPs elicited superior Pre-F-targeted immune responses in mice compared to conventional RSV pre-F antigen-encoding mRNA vaccines and pre-F-displaying nanoparticle formulations. Notably, the Pre-F-EABR vaccine induced more potent and broadly cross-reactive neutralizing antibodies, achieving both enhanced neutralization potency and significantly improved breadth against diverse RSV strains.

### Pre-F-EABR vaccine induces potent T-cell responses

**Fig 5:**
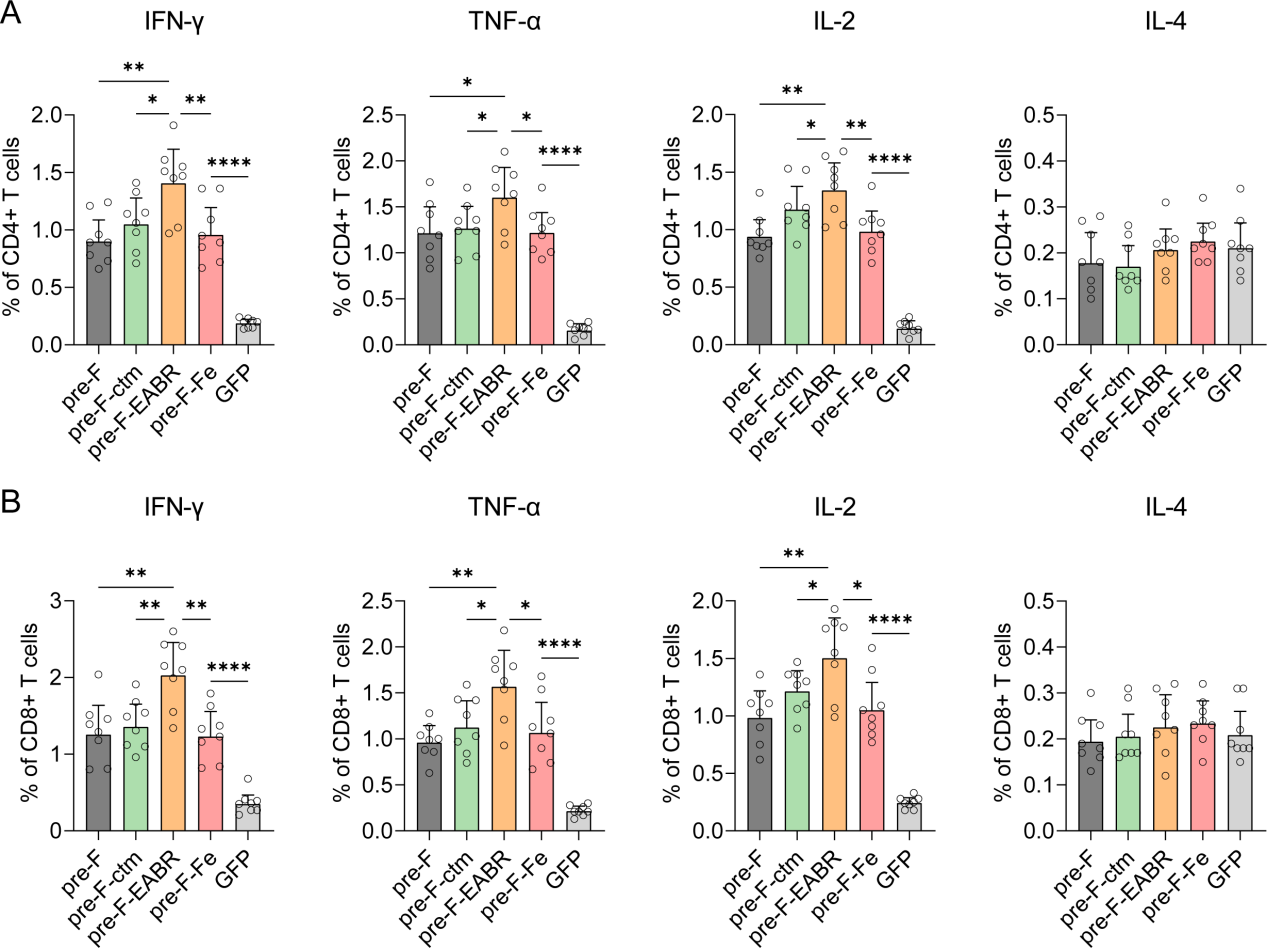
mRNA-LNP-induced cellular immune responses in BALB/c mice measured by ICS. Female BALB/c (*n* = 8 per group) were immunized as shown in Fig. 3A. Splenocytes were harvested on day 42 and stimulated with an RSV A2 F protein peptide pool. Flow cytometry was used to detect the immune response of mouse CD4+ (**A**) and CD8+ (**B**) T cells. The proportion of CD4+ T cells and CD8+ T cells expressing IFN-γ, TNF-α, IL-2, or IL-4 intracellular factors was shown. Data in the graph represent mean ± SD. Significant differences between groups were determined by a two-tailed unpaired *t*-test. *P < 0.05, **P < 0.01, ***P < 0.001, and ****P < 0.0001.

T-cell immune responses were evaluated in mice immunized with a 5 µg dose of mRNA-LNP vaccines in a prime-boost regimen (Fig. 3A) (40). Splenocytes were isolated 3 weeks after the boost immunization, stimulated with a peptide pool spanning the RSV A2 F protein, and analyzed by intracellular cytokine staining (ICS) (Fig. 5). The flow cytometry gating strategy for ICS is detailed in Fig. S3. Compared to the GFP mRNA-LNP control, all mRNA vaccines elicited robust CD4+ (Fig. 5A) and CD8+ (Fig. 5B) T-cell responses, as evidenced by the production of IFN-γ, TNF-α, and IL-2 cytokines (Fig. 5; Fig. S3B). These findings align with prior studies demonstrating that mRNA-LNP-encoded Pre-F antigens conferred strong CD4+ and CD8+ T-cell responses in mice (23, 33, 41). Of note, the Pre-F-EABR mRNA vaccine exhibited greater capacity to elicit T-cell immunity compared to control vaccines (Pre-F, Pre-F-ctm, and Pre-F-Fe mRNAs), as evidenced by significantly enhanced cytokine production profiles across both CD4+ and CD8+ T-cell subsets. Among CD4+ T cells, the Pre-F-EABR vaccination resulted in 1.3- to 1.6-fold increase in IFN-γ, ~1.3-fold higher TNF-α, and 1.1- to 1.4-fold enhanced IL-2 expression relative to control vaccines (Fig. 5A). Similarly, CD8+ T cells from Pre-F-EABR immunized mice showed 1.5- to 1.6-fold greater IFN-γ, 1.4- to 1.7-fold higher TNF-α, and 1.3- to 1.5-fold increased IL-2 expression compared to control groups (Fig. 5B). In contrast to our results, Hoffmann et al. reported comparable IFN-γ responses between their Spike-EABR mRNA vaccine and the conventional Spike mRNA vaccines (34). Remarkably, T-cell responses did not differ significantly between the Pre-F-Fe group and the Pre-F or Pre-F-ctm groups, indicating that ferritin incorporation into the DS-Cav1 construct did not alter cellular immunity. In contrast to the robust IFN-γ, TNF-α, and IL-2 responses, all mRNA-LNP vaccines elicited similarly low levels of IL-4 expression in both CD4+ (Fig. 5A) and CD8+ (Fig. 5B) T cells. The robust production of IFN-γ, TNF-α, and IL-2 alongside minimal IL-4 expression demonstrates that our tested mRNA-LNPs preferentially induce Th1-biased T-cell responses. Notably, the Pre-F-EABR vaccine elicited significantly higher levels of these Th1-associated cytokines compared to control vaccines, indicating a more pronounced Th1-skewed response. Collectively, these ICS results reveal that Pre-F-EABR mRNA-LNP activated potent Th1-oriented CD8+ and CD4+ T-cell responses that surpass those induced by the conventional DS-Cav1 mRNA vaccines, which likely contributes to the enhanced humoral immune responses.

**Fig 6:**
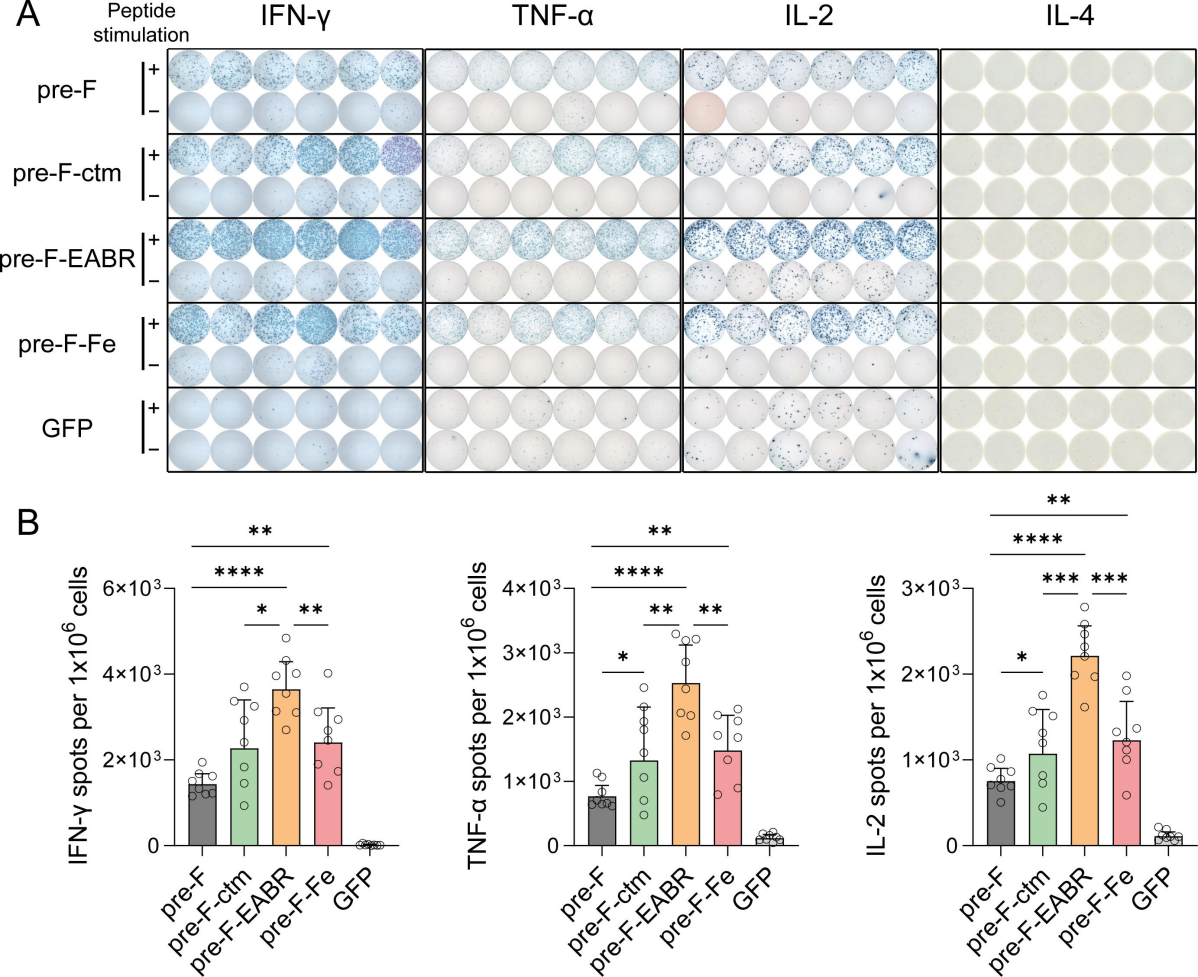
RSV F-specific T-cell responses characterized by ELISpot assays. (**A**) Female BALB/c mice (*n* = 8 per group) were immunized as described in Fig. 3A. Splenocytes were harvested 3 weeks post-boost immunization and stimulated with an RSV A2 F protein peptide pool (15-mers with 11-aa overlap). Representative images of spot-forming cells (SFCs) are shown. (**B**) Quantification of antigen-specific T cells secreting IFN-γ, TNF-α, IL-2, or IL-4. Data represent mean SFU per 10^6^ splenocytes ± SD. Statistical significance was determined by two-tailed unpaired *t*-test (*P < 0.05; **P < 0.01; ***P < 0.001; ****P < 0.0001).

Beyond the ICS analysis, we further assessed T-cell responses by ELISpot, quantifying IFN-γ, TNF-α, IL-2, and IL-4 secretion using the same splenocyte preparations (Fig. 6). Mirroring the ICS findings, the Pre-F-EABR mRNA vaccine elicited significantly stronger T-cell responses compared to control formulations. Specifically, the Pre-F-EABR vaccine induced 1.4- to 2.5-fold more IFN-γ-secreting cells, 1.7- to 3.3-fold more TNF-α-producing cells, and 1.7- to 2.9-fold more IL-2-secreting cells than controls, while IL-4 remained undetectable (Fig. 6B). These observations further support the robust Th1-skewed immune profile induced by the Pre-F-EABR vaccine. While ICS analysis revealed no statistically significant differences in T-cell responses between the Pre-F mRNA vaccine (encoding secreted DS-Cav1) and Pre-F-ctm mRNA vaccine (encoding membrane-anchored DS-Cav1) (Fig. 5), ELISpot assays showed significantly higher percentages of IL-2- and TNF-α-secreting T cells (*P* < 0.05 by two-tailed unpaired *t*-test; Fig. 6B). This finding aligns with previous ICS data demonstrating enhanced IL-2 and TNF-α production in CD4+ T cells in response to membrane-anchored DS-Cav1 versus the secreted form (33). This discrepancy may reflect the enhanced sensitivity of ELISpot in detecting low-frequency cytokine-secreting cells. Together, these findings demonstrate that the Pre-F-EABR mRNA vaccine effectively enhanced both the magnitude and polyfunctionality of T-cell responses in vaccinated mice.

### Pre-F-EABR vaccine provides enhanced protection against RSV challenge

**Fig 7:**
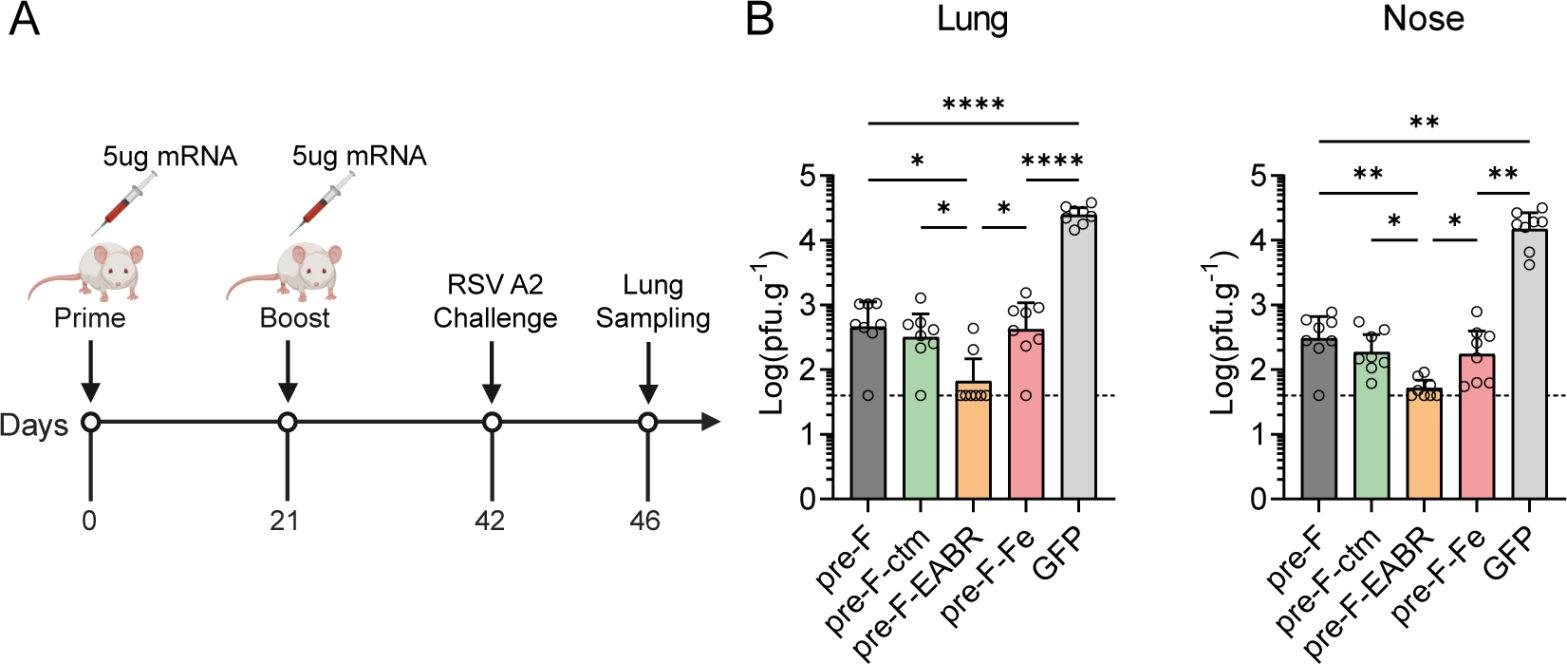
RSV challenge in immunized BALB/c mice. (**A**) Schematic of the immunization and RSV A2 challenge study in BALB/c mice (*n* = 8 per group). (**B**) Viral load quantification: RSV titers in lung (upper panel) and nasal (lower panel) tissue homogenates were measured by plaque assay at day 4 post-challenge. Data are presented as plaque-forming units per gram tissue (PFU/g; geometric mean ± 95% CI). The dashed horizontal line indicates the assay limit of detection. Statistical significance was determined by two-tailed unpaired *t*-test (*P < 0.05; **P < 0.01; ***P < 0.001; ****P < 0.0001).

To assess the protective efficacy of mRNA vaccines against RSV infection, BALB/c mice were vaccinated with 5 µg of mRNA-LNPs and challenged with RSV A2 3 weeks after the boost immunization (Fig. 7A). Four days post-challenge, lung and nasal tissues were harvested, and viral loads were quantified to assess vaccine-mediated protection efficacy. All tested RSV mRNA-LNP vaccines conferred significant protection against RSV A2 challenge, with markedly reduced viral titers in both lung and nose tissues (Fig. 7B). This protection profile correlated with the robust PreF-binding antibody responses, neutralizing activity, and T-cell immunity elicited by the vaccines (Fig. 3 to 6), and was consistent with previous reports (33, 41). Notably, the protective effect conferred by the Pre-F-EABR mRNA vaccine significantly outperformed other vaccine formulations, with viral loads reaching the limit of detection in both lung and nasal tissues (Fig. 7B). In contrast, mice receiving Pre-F, Pre-F-ctm, or Pre-F-Fe vaccines exhibited significantly higher viral loads in both lung (5.7-, 4.1-, and 5.7-fold increase, respectively; *P* < 0.05) and nasal tissue (7.2-, 4.3-, and 4.8-fold increase, respectively; *P* < 0.05) compared to the Pre-F-EABR group (Fig. 7B). This exceptional protection conferred by the Pre-F-EABR vaccine was consistent with its enhanced humoral and cellular immune responses.

## DISCUSSION

Herein, we designed an mRNA-based RSV vaccine, Pre-F-EABR, leveraging the recently reported EABR-based vaccine approach, and evaluated its immunogenicity and protective efficacy in mice. *In vitro* studies confirmed efficient production of eVLPs in cell culture supernatants following Pre-F-EABR mRNA transfection. Immunization with the Pre-F-EABR mRNA-LNP elicited significantly enhanced immune responses against both RSV A and B subtypes and conferred more efficient protection in mouse challenge studies. In comparison with the mRNA-LNP encoding Pre-F, the Pre-F-EABR construct elicited significantly more prominent RSV neutralizing antibody titers and robust T-cell responses. Notably, the viral loads in the lung and nose were also significantly lowered in mice that received the Pre-F-EABR mRNA-LNP (Fig. 7). Collectively, these results demonstrate the potential of Pre-F-EABR as a promising RSV vaccine candidate worthy of further development.

RNA-LNP-mediated vaccines, including those targeting RSV, are well-documented for their capacity to elicit robust cellular immune responses in both animal models and humans (20, 23, 32, 33, 40, 42, 43). In our study, the Pre-F-based mRNA vaccines also induced potent CD4+ and CD8+ T-cell immune responses in mice (Fig. 5 and 6), especially for the Pre-F-EABR vaccine, which elicited significantly stronger T-cell immune responses than the other candidates (Fig. 5). Mechanistically, the EABR platform not only facilitates antigen presentation on cell surfaces but also drives the assembly and release of eVLPs, mimicking the budding process of enveloped viruses (34). The secreted eVLPs may subsequently disseminate to lymph nodes distal from the injection site, thereby increasing the overall antigen exposure to the immune system and promoting more robust immune cell activation. This likely contributes to the observed enhancement in neutralizing antibody titers and T-cell responses elicited by the Pre-F-EABR vaccine. While correlates of protection against RSV infection remain incompletely defined, numerous studies have highlighted the critical roles of both neutralizing antibodies and cellular immune responses (27, 44–46). Consistent with this paradigm, Pre-F-EABR-vaccinated mice exhibited significantly reduced viral loads in the lung and nose after RSV challenge (Fig. 7), correlating with the elevated neutralizing activities and T-cell response profiles. However, further validation is needed to confirm *in vivo* eVLP assembly, and interactions between eVLPs and host immune cells also require further investigations. Such investigations will provide a more thorough insight into the mechanisms underlying the enhanced efficacy of the Pre-F-EABR mRNA vaccine.

Apart from older adults, there is an urgent need for vaccines that protect young infants against RSV-linked LTRD (1, 26). However, no pediatric RSV vaccines are currently accessible. The legacy of VAERD, which has been linked to Th2-biased immune response, remains a major obstacle in RSV pediatric vaccine development (26). In the BALB/c mouse model, IgG subclass profiles serve as key immunological markers, with IgG1 and IgG2a representing Th2- and Th1-associated responses, respectively, making the IgG2a/IgG1 ratio a hallmark of Th1/Th2 skewing (47). Our evaluation of IgG2a/IgG1 values demonstrated that all constructed mRNA-LNPs in this study induced balanced Th1/Th2 responses, though the Pre-F-EABR vaccine exhibited a comparatively higher IgG2a/IgG1 ratio compared to the Pre-F mRNA vaccine, suggesting a greater Th1 skewing (Fig. 3E). Unlike the IgG subclass results, ICS analysis demonstrated Th1-dominant immune responses across all RSV mRNA vaccine formulations, as evidenced by elevated expression of Th1-associated cytokines, including IFN-γ, TNF-α, and IL-2, and silenced expression of Th2-associated cytokine IL-4 (Fig. 5; Fig. S3B). This Th1-skewed cytokine profile was further validated by ELISpot assays, which not only confirmed the ICS findings but also revealed quantitatively enhanced, yet proportionally consistent, Th1-type response patterns (Fig. 6). Remarkably, the Pre-F-EABR vaccine induced significantly higher expression of IFN-γ, TNF-α, and IL-2 in both CD4+ and CD8+ T-cell populations compared to other vaccine formulations, indicating a more potent Th1-biased cellular immunity. This enhanced Th1 bias was further substantiated by ELISpot analysis, which showed significantly greater frequencies of IFN-γ+, TNF-α+, and IL-2+ T cells in Pre-F-EABR-vaccinated groups compared to controls (Fig. 6). The capacity of the Pre-F-EABR vaccine to elicit robust neutralizing antibody responses coupled with Th1-polarized cellular immunity suggests its potential as a candidate for pediatric RSV vaccine development. However, further preclinical studies are warranted to comprehensively assess the immunogenicity, protective efficacy, and safety profile of the Pre-F-EABR mRNA vaccine in proper animal models. This need is underscored by recent clinical trials of Moderna’s mRNA-1345 (RSV vaccine) and mRNA-1365 (RSV/human metapneumovirus combination vaccine), which reported increased incidence of severe respiratory disease among vaccinated young children ages 5 to 7 months (43). This finding underscores the complexities in pediatric RSV vaccine development and highlights the critical need for rational vaccine design coupled with rigorous preclinical assessment (43). Another key challenge in RSV mRNA vaccine development lies in achieving durable protection, as Moderna’s licensed *mResvia* vaccine demonstrated a significant decline in efficacy against RSV-associated LRTD in adults ages 60+ by 18 months post-vaccination (48). To address this limitation, future studies will focus on evaluating the longevity of immune protection conferred by the Pre-F-EABR mRNA vaccine and elucidating the mechanisms underlying its sustained efficacy.

Histopathological evaluation of RSV-challenged vaccinated mice in this study revealed a notable dissociation between virological and pathological outcomes. Histopathological examination of H&E-stained lung sections (Fig. S5) revealed similar pulmonary pathology profiles across all vaccinated groups, despite markedly different viral loads observed between the RSV mRNA vaccinated mice and the GFP control (Fig. 7B). These observations align with established literature demonstrating that BALB/c mice, despite their widespread use in RSV vaccine research, exhibit only semi-permissive characteristics for human RSV replication (49, 50). Previous studies have shown that even with high viral inoculum doses, this animal model typically exhibited minimal clinical disease manifestations and generally mild pulmonary pathology (50). To address this constraint, we have implemented a parallel experimental approach incorporating cotton rats (*Sigmodon hispidus*), which demonstrate enhanced permissiveness to RSV infection and more robust pathological manifestations (27, 33, 41, 51). These complementary studies are currently in progress, and we plan to report these additional findings—including any potential VAERD effects induced by the Pre-F-EABR vaccine—in future publications.

In this study, we have observed a dose-dependent humoral immune response elicited by the pre-F-EABR mRNA vaccine in BALB/c mice, with the 5 µg dosage consistently eliciting the highest serum antibody titers (Fig. S1A). Consequently, the 5 µg regimen was selected for subsequent immunization studies. Our selection is consistent with established mRNA vaccine research, where effective immunization doses in BALB/c mice typically fall within the 0.1–10 µg range (33, 34, 40, 41). However, when adjusted for body weight, these murine doses are substantially higher than the 30–100 µg doses used in human clinical trials for prophylactic mRNA vaccines (20, 30–32, 42, 43). To bridge this translational gap, future preclinical studies should evaluate the pre-F-EABR vaccine at clinically relevant doses in more physiologically appropriate models, such as non-human primates, which better recapitulate human RSV infection and immune responses.

In summary, we have developed a novel RSV mRNA vaccine that efficiently generates eVLPs. Our preclinical evaluation demonstrated that the Pre-F-EABR mRNA vaccine elicited significantly enhanced humoral responses and induced more robust T-cell responses in murine models. These compelling immunogenicity findings warrant further evaluation of this vaccine in additional preclinical models and subsequent clinical trials. This work provides critical insights into both the magnitude and quality of immune responses induced by this new and promising vaccine approach, offering valuable guidance for current and future RSV vaccine development programs.

## MATERIALS AND METHODS

### Viruses, cells, and antibodies

BHK-21 and HEK293T cells were cultured in Dulbecco’s modified Eagle’s medium (DMEM; BasalMedia, Shanghai, China) supplemented with 10% heat-inactivated fetal bovine serum (FBS; Sigma-Aldrich) and 10 U/mL penicillin-streptomycin (Gibco #15140122) at 37°C and 5% CO_2_. Expi293F cells were kindly provided by Prof. Xuepeng Wei from Guangzhou National Laboratory and cultured in Expi293 expression medium (Sino Biological, Shanghai, China) at 37°C and 8% CO_2_ with shaking at 135 rpm. All cell lines were confirmed to be mycoplasma-free using MycoBlue Mycoplasma Detector (Vazyme #D101-02). Antibodies used in this study include two anti-RSV F recombinant monoclonal antibodies, motavizumab (PDB: 3IXT) and D25 Fab (PDB: 6S3D), and an anti-GAPDH mouse monoclonal antibody (Proteintech #HRP-60004). Both motavizumab and D25 Fab were custom-ordered from Biointron (Nanjing, China). *Escherichia coli* DH5α cells (AlpalifeBio, Guangzhou, China) were used for plasmid cloning and were cultured in LB broth (Sango #A507002) supplemented with or without ampicillin (100 µg/mL) or kanamycin (50 µg/mL).

All RSV strains were propagated in HEp-2 cells in DMEM containing 2% FBS (D2). RSV A2 (ATCC VR-1540) was used for mouse challenge experiments. RSV A2, rRSV-A-0594-EGFP (ON1 genotype, rRSV-ON1-GFP) and rRSV-B-9671-EGFP (BA9 genotype, rRSV-BA9-GFP) were used in serum neutralization assays. Both rRSV-ON1-GFP and rRSV-BA9-GFP were generated using previously characterized optimized reverse genetics systems (39). Briefly, the pSMART BAC vector (1.6 µg) containing the full-length cDNA copy of the RSV-ON1-GFP or RSV-BA9-GFP antigenome was co-transfected with helper plasmids (pcDNA3.1(+) vector) encoding the corresponding N (1.6 µg), P (1.2 µg), M2-1 (0.8 µg), and L (0.4 µg) open reading frames into HEp-2 cells with Neon Transfection System (Thermo Fisher Scientific). Prior to the electroporation, HEp-2 cells were infected with MVA-T7 (52) (MOI = 5) for 1 h at 37°C. Electroporated cells were seeded in 6-well plates (1 × 10^6^ cells/well) and monitored for fluorescent cell foci or syncytium formation. Rescued rRSVs were harvested in 5–6 days post-transfection by two freeze-thaw cycles. Viral titers were determined by plaque assays. Briefly, virus stocks were serially diluted in D2 and added in duplicate to 90% confluent HEp-2 cell monolayers in 12-well plates. After 2–4 h incubation at 37°C, 3 mL of 0.75% carboxymethyl cellulose solution (prepared in D2) was added per well. Following 5 days of incubation at 37°C, cells were fixed with 4% paraformaldehyde (PFA) and stained with the Crystal Violet Staining Solution (Beyotime #C0121). Viral plaques were counted, and titers (PFU/mL) were determined.

### mRNA synthesis

RSV Pre-F-expressing mRNAs were designed based on the Ds-Cav1-stabilized F protein derived from the A2 strain (14). The T4-fibritin trimerization domain (foldon) and amino acid substitutions were incorporated as shown in Fig. 1A. Specifically, mRNA encoding Pre-F-EABR was designed by fusing the Ds-Cav1 sequence to the transmembrane domain (residues 514–551 of RSV F from A2 strain), the EPM motif (residues 243–290 of mouse FcgRII-B1), and the EABR domain (residues 160–217 of human CEP55 protein). The EPM and EABR motifs were separated by a 4-residue GS linker. The Pre-F-Fe encoding sequence was generated by fusing the Ds-Cav1 sequence (without foldon) to *H. pylori* ferritin DNA, separated by a 3-residue GS linker inserted between them. All sequences were codon-optimized and integrated into our mRNA production plasmid containing the T7 RNA polymerase promoter, 5′ UTR, open reading frame, and 3′ UTR. Linearized DNA templates were PCR-amplified using Phanta Flash Master Mix (Vazyme #P510-03), gel-purified, and extracted with the GeneJET Gel Extraction Kit (Thermo Fisher Scientific #K0692). mRNAs were synthesized by T7 RNA polymerase-mediated *in vitro* transcription reaction using an Enzyme Mix (Hzymes Biotech #HBP000331-2), the ribonucleoside triphosphates mix (100 mM each: ATP, GTP, CTP, and N1-methyl-pseudouridine triphosphate; Henovcom #HN3004), and the Cap1 analog LZCap (Henovcom #HN3004). Post-IVT, DNA templates were digested by DNase I (NEB #M0303L), and mRNAs were purified by LiCl precipitation. After dissolution in DNase/RNase-free water (Beyotime #ST876), concentrations were quantified spectrophotometrically. mRNA integrity was verified by agarose gel electrophoresis and capillary electrophoresis (Agilent 5200 Fragment Analyzer, DNF-471 kit). Data were analyzed using ProSize software (Agilent). All mRNAs were stored at −80°C.

### LNP encapsulation of mRNAs

The mRNAs were encapsulated in LNPs via microfluidic mixing (RNACure) by combining the mRNA and lipid solutions at a 3:1 vol ratio and a flow rate of 12 mL/min. Specifically, mRNAs were diluted in 50 mM citrate buffer (pH 4.0) to 150 µg/mL, while the lipid mixture—consisting of SM-102 ionizable lipid (AVT, Shanghai, #O02010), distearoylphosphatidylcholine (DSPC; AVT #S01005), cholesterol (AVT #57-88-5), and poly(ethylene glycol)2000-dimyristoylglycerol (PEG2000-DMG; AVT #O02005) in a 50:38.5:10:1.5 molar ratio—was dissolved in ethanol to 8 mM concentration. The resulting LNPs were dialyzed against PBS to remove ethanol and displace the acidic solution. LNPs were characterized by measuring the hydrodynamic size and polydispersity index by dynamic light scattering (Malvern Nano-ZS zetasizer), while RNA concentration and encapsulation efficiency were determined using the Quant-iT RiboGreen assay (Thermo Fisher Scientific) following the manufacturer’s protocol.

### Cryo-EM characterization of mRNA-LNP

For cryo-EM microscopy, Quantifoil R1.2/1.3 300-mesh holy carbon gold grids with 2 nm ultrathin carbon support films were glow-discharged prior to sample application. A 3 µL aliquot of mRNA-LNP solution was transferred onto each grid and incubated for 1 min at room temperature. Excess solution was blotted for 3 s in a Vitrobot Mark IV (Thermo Fisher Scientific) chamber maintained at 4°C and 100% humidity, followed by rapid plunging into liquid ethane. Cryo-preserved grids were loaded into a 300 kV Titan Krios G4 microscope (Thermo Fisher Scientific) equipped with a Selectris-X energy filter (10 eV slit width) and Falcon 4i direct electron detector. Data collection was performed using EPU2 software in counting mode at 1.94 Å/pixel resolution, with 3–4 μm defocus and a total electron dose of 20 e/Å^2^.

### mRNA transfection and protein expression

BHK-21 cells were seeded in 12-well plates and incubated at 37°C overnight to reach 70%–90% confluency at transfection. mRNAs were complexed with Lipo8000 Transfection Reagent (Beyotime #C0533) in Opti-MEM I Reduced Serum Medium (Gibco #31985070) according to the manufacturer’s protocol. For western blot analysis, samples were mixed with LDS buffer (Thermo Fisher #NP0008) and resolved on 4%–20% FuturePAGE precast gels (ACE Biotechnology, Changzhou, China). Proteins were transferred to PVDF membrane (Millipore #IPVH00010) and probed overnight at 4°C with either anti-RSV F (motavizumab) or anti-GAPDH antibodies. After washing, membranes were incubated for 1 h at room temperature with HRP-conjugated goat anti-human IgG (Beyotime #A0201) or anti-mouse IgG (Sangon #D110087), followed by detection using eECL Western Blot Kit (CoWin Biotech, Jiangsu, China, #CW0049S) on an iBright CL750 Imaging System (Thermo Fisher Scientific). For immunofluorescence detection of RSV Pre-F protein expression, transfected cells were fixed with 4% PFA at 24 h post-transfection and washed with PBS. Fixed cells were permeabilized with 0.2% Triton X-100, then blocked with 5% BSA for 30 min at room temperature. Following blocking, cells were incubated with D25 Fab (1 µg/mL in PBS) for 1 h at 37°C, then stained with Alexa Fluor 488-conjugated goat anti-human IgG (1:2,000 dilution in PBS, Thermo Fisher #A48276) for 1 h at 37°C. For cell surface antigen detection, the protocol was modified by omitting the Triton X-100 permeabilization step while maintaining all other conditions. Fluorescence imaging was performed using an Operetta CLS high-content analysis system (PerkinElmer) following final PBS washes.

### Production of EABR eVLPs

The production and purification of eVLPs followed a previously described protocol (34) with minor modifications. HEK293T cells were seeded in 10 cm dishes (Corning #430167) and incubated at 37°C overnight to reach 70%–90% confluency at transfection. Each dish was transfected with 10 µg mRNA with Lipo8000 Transfection Reagent in Opti-MEM I Reduced Serum Medium. Following 72 h incubation at 37°C, cell supernatants were collected, centrifuged, filtered through 0.45 µm membranes, and concentrated using Amicon Ultra-15 centrifugal filters (100 kDa MWCO; Millipore). The concentrated samples were then ultracentrifuged at 135,000 × *g* for 2 h at 4°C over a 20% wt/vol sucrose cushion using a Sorvall WX100+ ultracentrifuge (Thermo Fisher Scientific). Supernatants were removed, and pellets were re-suspended in 200 µL sterile PBS at 4°C overnight. Residual cell debris was removed by centrifugation at 10,000 × *g* for 10 min, and the final supernatants were collected for analysis. eVLP production was confirmed by western blot. The production of eVLPs was also tested in Expi293F cells. The day before transfection, Expi293F cells were seeded at a density of 2 × 10^6^ cells/mL in Expi293 expression medium and adjusted to 2.5 × 10^6^ cells/mL at transfection. Cells were transfected with 2 µg mRNA per milliliter culture using Lipo8000 Transfection Reagent. Seventy-two hours post-transfection, supernatants were collected, and eVLPs were purified using the same protocol as for HEK293T cells.

### Mouse immunization

All mouse immunization procedures were conducted in accordance with the approved protocol (GZLAB-AUCP-2022-10-A02) from the Animal Care and Use Committee of Guangzhou National Laboratory. Female BALB/c mice (5–7 weeks old; GemPharmatech, Guangzhou, China) were divided into groups (*n* = 5, 8, or 16) and received two intramuscular immunizations (0.1, 1, or 5 µg mRNA-LNP in 100 µL) in the hind leg at 3-week intervals. The Pfizer subunit vaccine (RSVpreF; ABRYSVO) (18, 36, 37) was provided as a gift by Professor Jinzhong Lin’s group at Fudan University, who purchased it from the pharmacy, and a dose of 1 µg was administered to each mouse. Blood samples were collected via retro-orbital bleeding under 2%–3% isoflurane anesthesia 21 days post-each immunization for antibody titer determination. For ICS assays and ELISpot assays, mice were euthanized by cervical dislocation under 2%–3% isoflurane anesthesia 21 days post-boost, followed by aseptic spleen collection.

### Enzyme-linked immunosorbent assay

ELISAs were performed to quantify serum antibody titers against RSV Pre-F or post-F proteins. Ninety-six-well ELISA plates (Corning #9018) were coated overnight at 4°C with 1 µg/mL recombinant RSV Pre-F (Sino Biological #11049-VNAS) or Post-F (Sino Biological #11049-V08H5) in carbonate/bicarbonate buffer. After washing with PBS containing 0.05% Tween-20 (PBS-T), plates were blocked for 1 h at 37°C with 5% non-fat milk in PBS. Mouse sera were initially diluted 100-fold, followed by threefold serial dilutions. Diluted samples were incubated on coated plates for 1 h at 37°C, washed three times with PBS-T, and then incubated with HRP-conjugated goat anti-mouse IgG (1:2,000; Sangon #D110058) for 1 h at 37°C. Following additional washes, reactions were developed with TMB substrate solution (Beyotime #P0209) for 15 min at 37°C and stopped with stop solution (Beyotime #P0215). Absorbance was measured at 450 nm using an Ensight Multimode Plate Reader (PerkinElmer). Endpoint titers were defined as the highest reciprocal dilution yielding an optical density ≥2× background. For IgG subclass analysis, identical procedures were performed using HRP-conjugated goat anti-mouse IgG1 (Abcam #ab97240) or IgG2a (Abcam #ab97245).

### Neutralization assays

Serum neutralization assays were conducted as previously described (21, 27, 53, 54) with modifications. Briefly, sera were heat-inactivated at 56°C for 30 min and serially diluted (initial 1:50 dilution, followed by threefold dilutions in D2 medium) in 96-well plates. Equal volumes of diluted sera were mixed with RSV at optimized titers (A2/ON1: 800 PFU/well; BA9: 600 PFU/well) and incubated at 37°C for 1 h. The serum-virus mixtures were then transferred to HEp-2 cell monolayers (3 × 10^4^ cells/well in 96-well plates, seeded 24 h prior) and incubated at 37°C for 30 h before PBS washing and fixation (4% PFA). For RSV A2 neutralization, fixed cells were processed for immunostaining. Briefly, fixed cells were permeabilized with 0.2% Triton X-100, then blocked with 5% BSA for 30 min at room temperature. After blocking, cells were incubated with D25 Fab (1 µg/mL in PBS) for 1 h at 37°C, followed by staining with Alexa Fluor 488-conjugated goat anti-human IgG (1:2,000 dilution in PBS) for 1 h at 37°C. Between each step, cells were washed three times with PBS. GFP fluorescence was quantified directly for rRSV-ON1-GFP and rRSV-BA9-GFP strains. All plates were analyzed with an Operetta CLS high-content analysis system, and neutralization titers were calculated using four-parameter non-linear regression analysis (GraphPad Prism 6). A representative picture of the RSV A2 neutralizing assay is shown in Fig. S4.

### Intracellular cytokine staining

Mouse spleens were homogenized through a 40 µm cell strainer using syringe plungers in 5 mL of complete I10 medium (Iscove’s Modified Dulbecco’s Medium, Gibco #12440053) supplemented with 10% FBS, 50 µM β-mercaptoethanol, and 1 U/mL penicillin-streptomycin. After centrifugation, erythrocytes were lysed using 5 mL ACK lysis buffer (Beyotime #C3702) for 3 min at room temperature, followed by neutralization with 10 volumes of PBS. The resulting splenocytes were filtered through 40 µm strainers, pelleted, and resuspended in 2 mL I10 medium. The splenocytes were then enumerated, and cell concentrations were adjusted to ~1 × 10^7^/mL for subsequent assays.

For intracellular cytokine staining, 100 μL of splenocytes were stimulated in 96-well U-bottom plates with an equal volume of RSV A2 F0 peptide pool (15-mers overlapping by 11 amino acids, 1  µg/mL in I10, Sino Biological) or control treatments (DMSO vehicle or 1 µg/mL concanavalin A; InvivoGen #inh-cona) for 1 h at 37°C. Protein transport inhibitors (GolgiStop/GolgiPlug, BD Biosciences #554724/550583; 1:200 dilution in I10) were then added (50 µL/well), followed by 7 h incubation at 37°C.

Following two PBS washes, cells were sequentially stained with Fixable Viability Stain 780 (BD #565388) for 15 min at room temperature, then with a surface marker antibody cocktail (all antibodies diluted 1:100 in MACS buffer [PBS with 2% BSA and 1 mM EDTA]) containing: anti-CD3e FITC (BD #553061), anti-CD4 PE-Cy7 (BD #552775), and anti-CD8a BV510 (BD #563068) for 30 min at 4°C in the dark. Following washing with MACS buffer, cells were fixed and permeabilized with Cytofix/Cytoperm solution (BD #554722, 100 µL/well, 20 min, 4°C).

For intracellular cytokine detection, cells were washed with Perm/Wash buffer (BD #554723) and stained with a panel of anti-cytokine antibodies (all 1:100 diluted in Perm/Wash buffer), including anti-IFN-γ BV711 (BD #564336), anti-TNF BB700 (BD #566510), anti-IL-2 PE (BD #554428), and anti-IL-4 BV786 (BD #564006), for 30 min at 4°C in the dark. After a final Perm/Wash buffer wash, cells were resuspended in 200 µL MACS buffer and acquired on a Novocyte Advanteon flow cytometer (Agilent Technologies). Data analysis was performed using NovoExpress software, with the gating strategy and representative results shown in Fig. S3.

### ELISpot assay

ELISpot assays were performed to quantify F protein-specific T-cell responses by measuring IFN-γ, TNF-α, IL-2, and IL-4 secretion in splenocytes obtained from the above ICS experiments. Cells were stimulated for 30 h with either RSV A2 F0 peptide pool (15-mers overlapping by 11 amino acids at 1 µg/mL in I10), DMSO vehicle control (0.1%), or concanavalin A (1 µg/mL positive control). Cytokine production was detected using mouse ELISpot Plus kits (MabTech: IFN-γ #3321-4HST-2, IL-2 #3441-4HPW-2, TNF-α #3511-4HPW-2, and IL-4 #3311-4HPW-2) according to the manufacturer’s instructions. Spot-forming units (SFU) were quantified using an automated ELISpot reader, normalized to SFU per 10⁶ viable cells. Background levels were calculated as the 95% percentile of the SFU observed in non-stimulated splenocytes.

### RSV challenge

The RSV challenge study was approved by the Animal Care and Use Committee of Guangzhou National Laboratory (GZLAB-AUCP-2023-10-A7) and conducted under ABSL2 conditions. Female BALB/c mice (*n* = 8 per group, aged 5–7 weeks, GemPharmatech) were immunized twice I.M. with 5 µg mRNA at 3-week intervals. Three weeks post-boost immunization, mice were anesthetized under isoflurane (2%–3%) and intranasally challenged with 1 × 10^6^ PFU RSV A2 in 50 µL, while control groups received PBS immunization and mock challenged. Four days post-infection, mice were euthanized by cervical dislocation under isoflurane anesthesia (2%–3%). Lungs and nose were then harvested, homogenized, and clarified by centrifugation (3,000 × *g*, 10 min). Viral loads were assessed via plaque assays on HEp-2 cell monolayers in 12-well plates and expressed as PFU/g.

### Statistical analysis

Statistical analyses were conducted using GraphPad Prism 6.0. Antibody responses (ELISA and neutralization assays, Fig. 3 to 4; Fig. S1 and S2) and respiratory tract viral titers (Fig. 7) were expressed as geometric mean with 95% confidence intervals (95% CI). Cellular immune responses, including cytokine production (ICS, Fig. 5) and antigen-specific T-cell frequencies (ELISpot, Fig. 6), were expressed as mean ± standard deviation (SD). Differences between immunized groups were statistically evaluated using the two-tailed unpaired *t*-test that passed the Anderson-Darling normality test or by the Mann-Whitney test that included non-parametric populations. Statistical significance was determined by a *P* value below 0.05, and the significant differences are indicated with asterisks in each graph (*P < 0.05; **P ≤ 0.01; ***P ≤ 0.001; ****P ≤ 0.0001).

## Data Availability

The data that support the findings of this study are available upon reasonable request.

## SUPPLEMENTAL MATERIAL

Supplemental material jvi.01209-25-s0001.docx Figures S1 to S5 and amino acid sequences.

ASM does not own the copyrights to Supplemental Material that may be linked to, or accessed through, an article. The authors have granted ASM a non-exclusive, world-wide license to publish the Supplemental Material files. Please contact the corresponding author directly for reuse.
