# Supplementary material for: Developing an eVLP mRNA vaccine for respiratory syncytial virus with enhanced pre-fusion targeting humoral responses
Source: J Virol. 2025 Sep 30;99(10):e01209-25. doi: 10.1128/jvi.01209-25 (PMC12548431; doi:10.1128/jvi.01209-25)
Supplement: Supplemental material — Figures S1 to S5 and amino acid sequences. [file jvi.01209-25-s0001.docx]

Supplementary information


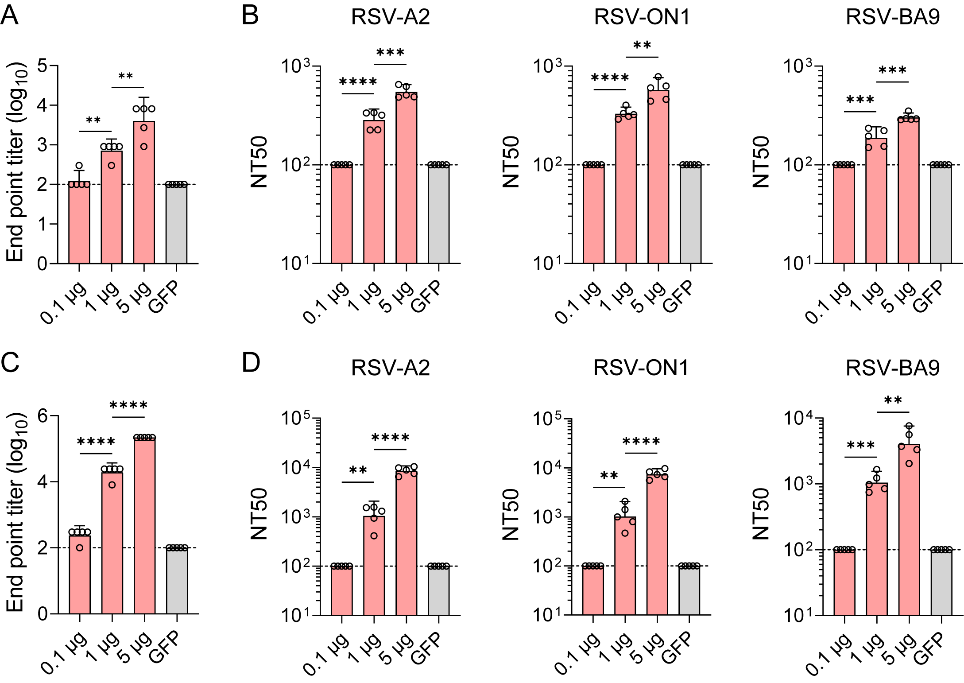


**Supplementary Figure 1. Dose-dependent immune responses induced by pre-F-EABR mRNA-LNP vaccination in mice.** Female BALB/c mice (n = 5 per group) were immunized I.M. with 0.1, 1, or 5 μg doses of pre-F-EABR mRNA-LNP vaccines in a prime-boost regimen (days 0 and 21). RSV pre-F-specific IgG titers measured by ELISA at (**A**) day 21 (post-prime) and (**C**) day 42 (post-boost). Neutralization activity was assessed against RSV-A2 (right panel), RSV-ON1-GFP (middle panel), and RSV-BA9-GFP (left panel) strains at (**B**) day 21 and (**D**) day 42. NT50 values (reciprocal serum dilution achieving 50% neutralization) were determined by four-parameter sigmoidal curve fitting. Data represent geometric mean ± 95% CI (dotted line indicates limit of detection). Statistical significance was calculated by two-tailed unpaired t-test (**p* < 0.05; ***p* < 0.01; ****p* < 0.001; *****p* < 0.0001).


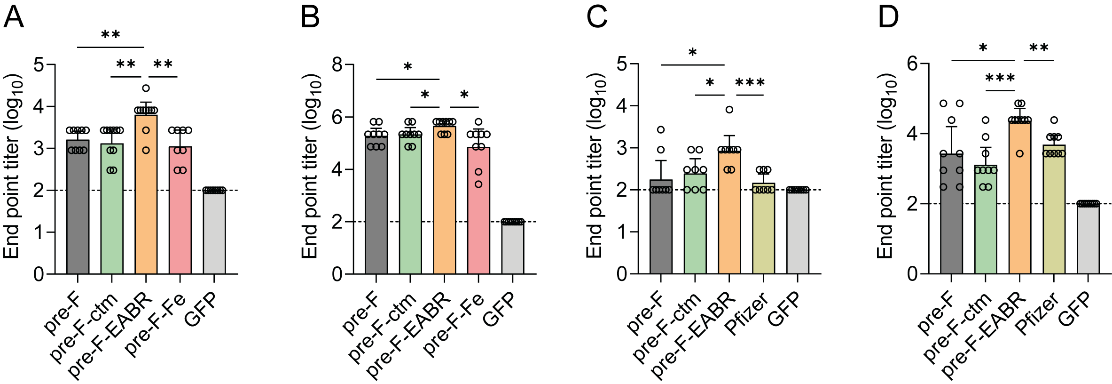


**Supplementary Figure 2. RSV pre-F-specific IgG antibody responses induced by 1 μg and 5 μg mRNA-LNPs (including the pre-F-ctm mRNA-LNP) in BALB/c mice.** Serum IgG titers against RSV pre-F were measured by ELISA following immunization with either 5 μg (**A**, **B**) or 1 μg (**C**, **D**) mRNA-LNPs (including pre-F-ctm construct) in a prime-boost regimen. Results show IgG titers at (**A**, **C**) day 21 (post-prime) and (**B**, **D**) day 42 (post-boost). Data represent geometric mean ± 95% CI (dotted line indicates limit of detection). Statistical significance was calculated by two-tailed unpaired *t*-test (**p* < 0.05; ***p* < 0.01; ****p* < 0.001; *****p* < 0.0001).


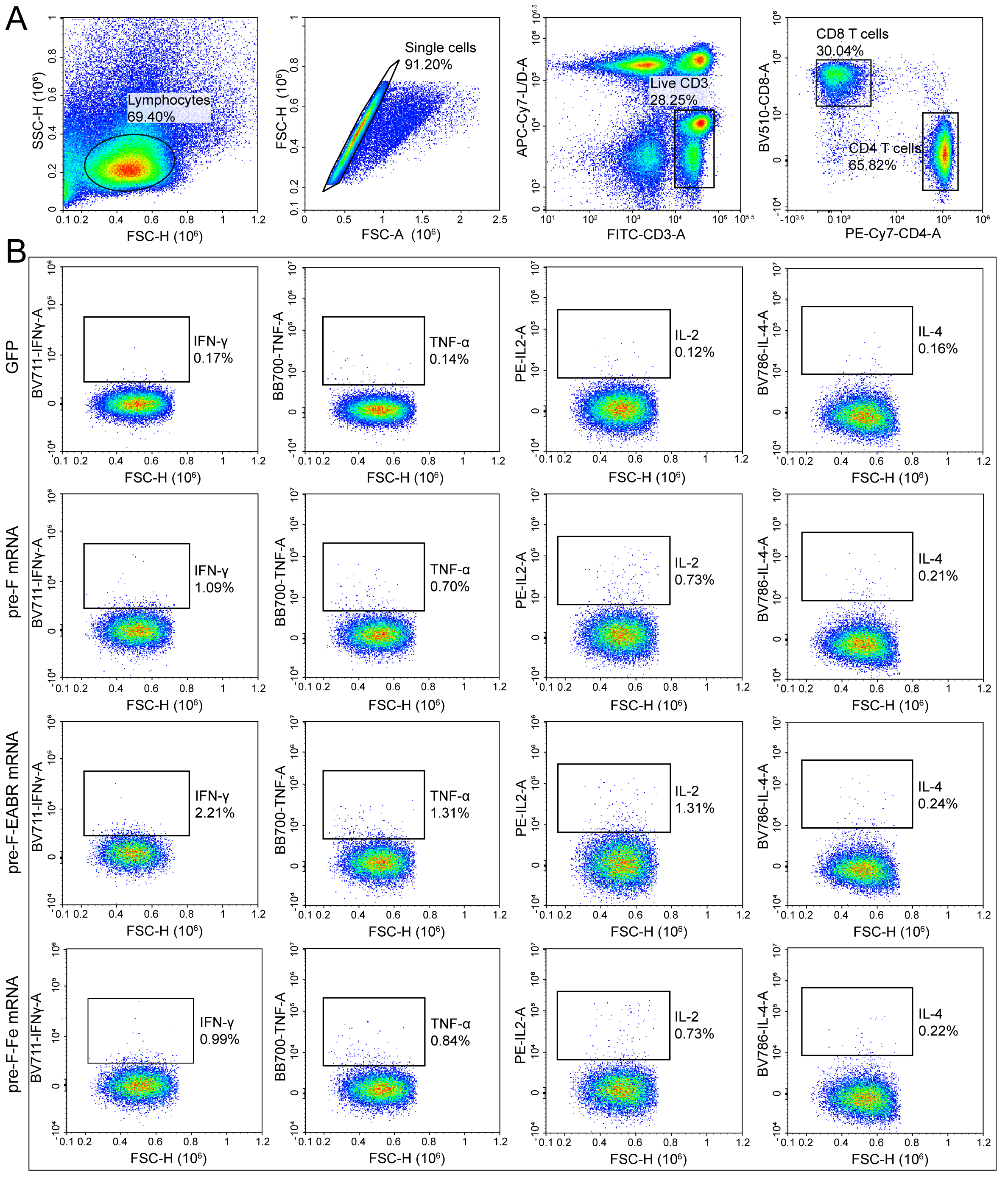


**Supplementary Figure 3. Flow cytometry gating strategy for intracellular cytokine staining analysis related to Figure 5.** (**A**, **B**) Schematic representation of the gating hierarchy used for intracellular cytokine staining of mouse splenocytes. (**B**) Representative examples demonstrating CD4+ T cell cytokine response (IFN-γ, TNF-α, IL-2, and IL-4) is shown for an individual mouse from each vaccinated group. CD8+ T cell responses were analyzed using an identical gating strategy (data not shown).


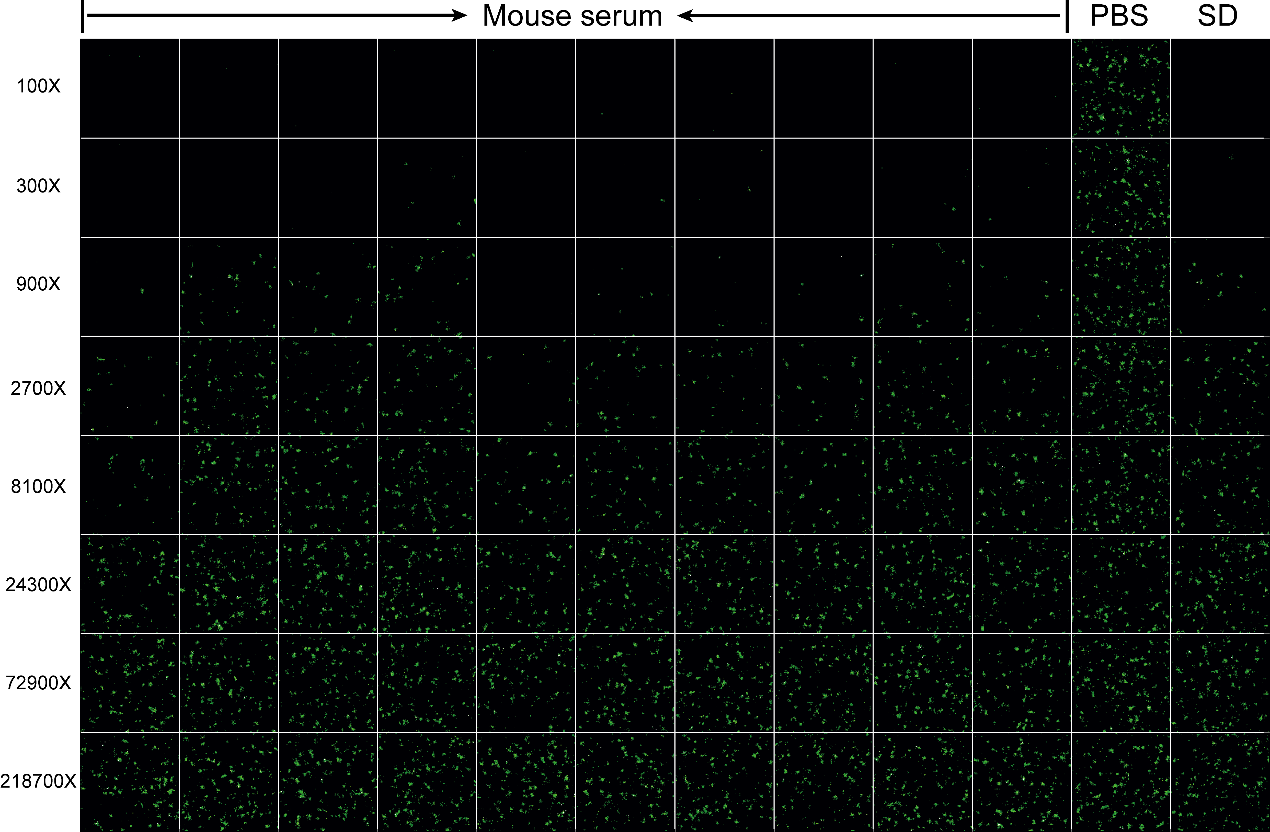


**Supplementary Figure 4. Schematic representation of the RSV microneutralization assay.** Image was acquired using the Operetta CLS high-content analysis system (10× magnification), with standardized serum (SD) serving as the positive neutralization control.


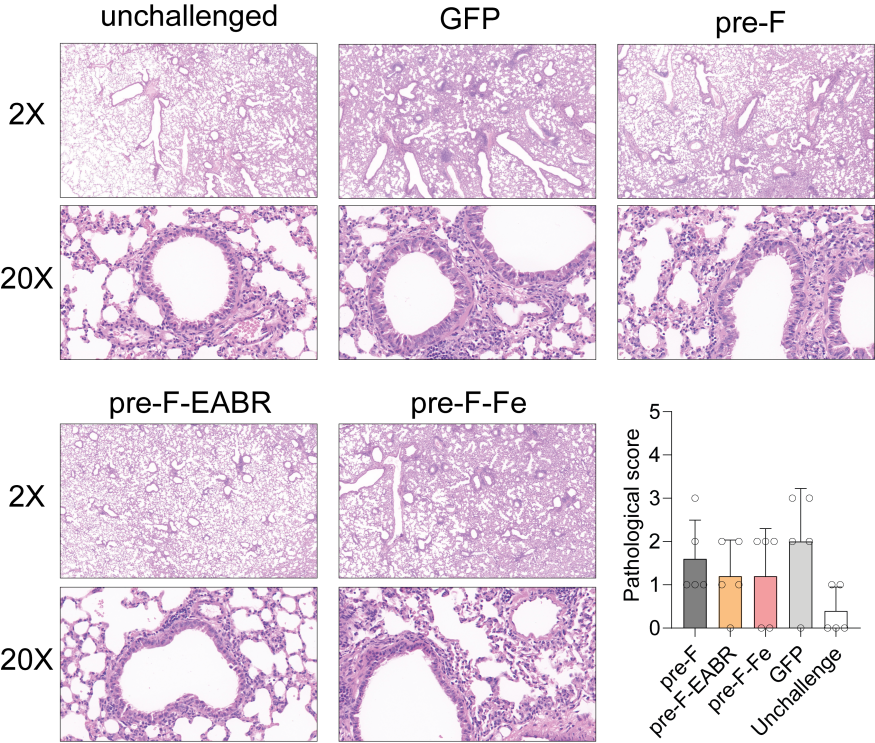


**Supplementary Figure 5. Lung pathology following RSV A2 challenge in vaccinated BALB/c mice** (corresponding to Figure 7). Representative hematoxylin and eosin (H&E)-stained lung sections are presented alongside quantitative pathology scoring from RSV A2-challenged mice.

**Amino acid sequence for mRNA vaccine design:**

**Pre-F:** MELLILKANAITTILTAVTFCFASGQNITEEFYQSTCSAVSKGYLSALRTGWYTSVITIELSNIKENKCNGTDAKVKLIKQELDKYKNAVTELQLLMQSTPPTNNRARRELPRFMNYTLNNAKKTNVTLSKKRKRRFLGFLLGVGSAIASGVAVCKVLHLEGEVNKIKSALLSTNKAVVSLSNGVSVLTFKVLDLKNYIDKQLLPILNKQSCSISNIETVIEFQQKNNRLLEITREFSVNAGVTTPVSTYMLTNSELLSLINDMPITNDQKKLMSNNVQIVRQQSYSIMCIIKEEVLAYVVQLPLYGVIDTPCWKLHTSPLCTTNTKEGSNICLTRTDRGWYCDNAGSVSFFPQAETCKVQSNRVFCDTMNSLTLPSEINLCNVDIFNPKYDCKIMTSKTDVSSSVITSLGAIVSCYGKTKCTASNKNRGIIKTFSNGCDYVSNKGMDTVSVGNTLYYVNKQEGKSLYVKGEPIINFYDPLVFPSDEFDASISQVNEKINQSLAFIRKSDELLSAIGGYIPEAPRDGQAYVRKDGEWVLLSTFL

**Pre-F-ctm:** MELLILKANAITTILTAVTFCFASGQNITEEFYQSTCSAVSKGYLSALRTGWYTSVITIELSNIKENKCNGTDAKVKLIKQELDKYKNAVTELQLLMQSTPPTNNRARRELPRFMNYTLNNAKKTNVTLSKKRKRRFLGFLLGVGSAIASGVAVCKVLHLEGEVNKIKSALLSTNKAVVSLSNGVSVLTFKVLDLKNYIDKQLLPILNKQSCSISNIETVIEFQQKNNRLLEITREFSVNAGVTTPVSTYMLTNSELLSLINDMPITNDQKKLMSNNVQIVRQQSYSIMCIIKEEVLAYVVQLPLYGVIDTPCWKLHTSPLCTTNTKEGSNICLTRTDRGWYCDNAGSVSFFPQAETCKVQSNRVFCDTMNSLTLPSEINLCNVDIFNPKYDCKIMTSKTDVSSSVITSLGAIVSCYGKTKCTASNKNRGIIKTFSNGCDYVSNKGMDTVSVGNTLYYVNKQEGKSLYVKGEPIINFYDPLVFPSDEFDASISQVNEKINQSLAFIRKSDELLHNVNAGKSTTNIMITTIIIVIIVILLSLIAVGLLLYCKARSTPVTLSKDQLSGINNIAFSN

**Pre-F-EABR:** MELLILKANAITTILTAVTFCFASGQNITEEFYQSTCSAVSKGYLSALRTGWYTSVITIELSNIKENKCNGTDAKVKLIKQELDKYKNAVTELQLLMQSTPPTNNRARRELPRFMNYTLNNAKKTNVTLSKKRKRRFLGFLLGVGSAIASGVAVCKVLHLEGEVNKIKSALLSTNKAVVSLSNGVSVLTFKVLDLKNYIDKQLLPILNKQSCSISNIETVIEFQQKNNRLLEITREFSVNAGVTTPVSTYMLTNSELLSLINDMPITNDQKKLMSNNVQIVRQQSYSIMCIIKEEVLAYVVQLPLYGVIDTPCWKLHTSPLCTTNTKEGSNICLTRTDRGWYCDNAGSVSFFPQAETCKVQSNRVFCDTMNSLTLPSEINLCNVDIFNPKYDCKIMTSKTDVSSSVITSLGAIVSCYGKTKCTASNKNRGIIKTFSNGCDYVSNKGMDTVSVGNTLYYVNKQEGKSLYVKGEPIINFYDPLVFPSDEFDASISQVNEKINQSLAFIRKSDELLSAIGGYIPEAPRDGQAYVRKDGEWVLLSTFLHNVNAGKSTTNIMITTIIIVIIVILLSLIAVGLLLYCKALPGNPDHREMGETLPEEVGEYRQPSGGSVPVSPGPPSGLEPTSSSPYGGGSFNSSINNIHEMEIQLKDALEKNQQWLVYDQQREVYVKGLLAKIFELEKKTETAAHSLP

**Pre-F-Fe:** MELLILKANAITTILTAVTFCFASGQNITEEFYQSTCSAVSKGYLSALRTGWYTSVITIELSNIKENKCNGTDAKVKLIKQELDKYKNAVTELQLLMQSTPPTNNRARRELPRFMNYTLNNAKKTNVTLSKKRKRRFLGFLLGVGSAIASGVAVCKVLHLEGEVNKIKSALLSTNKAVVSLSNGVSVLTFKVLDLKNYIDKQLLPILNKQSCSISNIETVIEFQQKNNRLLEITREFSVNAGVTTPVSTYMLTNSELLSLINDMPITNDQKKLMSNNVQIVRQQSYSIMCIIKEEVLAYVVQLPLYGVIDTPCWKLHTSPLCTTNTKEGSNICLTRTDRGWYCDNAGSVSFFPQAETCKVQSNRVFCDTMNSLTLPSEINLCNVDIFNPKYDCKIMTSKTDVSSSVITSLGAIVSCYGKTKCTASNKNRGIIKTFSNGCDYVSNKGMDTVSVGNTLYYVNKQEGKSLYVKGEPIINFYDPLVFPSDEFDASISQVNEKINQSLAFIRKSDELLGSGGSGDIIKLLNEQVNKEMQSSNLYMSMSSWCYTHSLDGAGLFLFDHAAEEYEHAKKLIIFLNENNVPVQLTSISAPEHKFEGLTQIFQKAYEHEQHISESINNIVDHAIKSKDHATFNFLQWYVAEQHEEEVLFKDILDKIELIGNENHGLYLADQYVKGIAKSRKS
